# Measuring the Effect of Inter-Study Variability on Estimating Prediction Error

**DOI:** 10.1371/journal.pone.0110840

**Published:** 2014-10-17

**Authors:** Shuyi Ma, Jaeyun Sung, Andrew T. Magis, Yuliang Wang, Donald Geman, Nathan D. Price

**Affiliations:** 1 Institute for Systems Biology, Seattle, Washington, United States of America; 2 Department of Chemical and Biomolecular Engineering, University of Illinois, Urbana, Illinois, United States of America; 3 Asia Pacific Center for Theoretical Physics, Pohang, Gyeongbuk, Republic of Korea; 4 Center for Biophysics and Computational Biology, University of Illinois, Urbana, Illinois, United States of America; 5 Sage Bionetworks, Seattle, Washington, United States of America; 6 Institute for Computational Medicine & Department of Applied Mathematics and Statistics, John Hopkins University, Baltimore, Maryland, United States of America; National Taiwan University, Taiwan

## Abstract

**Background:**

The biomarker discovery field is replete with molecular signatures that have not translated into the clinic despite ostensibly promising performance in predicting disease phenotypes. One widely cited reason is lack of classification consistency, largely due to failure to maintain performance from study to study. This failure is widely attributed to variability in data collected for the same phenotype among disparate studies, due to technical factors unrelated to phenotypes (e.g., laboratory settings resulting in “batch-effects”) and non-phenotype-associated biological variation in the underlying populations. These sources of variability persist in new data collection technologies.

**Methods:**

Here we quantify the impact of these combined “study-effects” on a disease signature’s predictive performance by comparing two types of validation methods: ordinary randomized cross-validation (RCV), which extracts random subsets of samples for testing, and inter-study validation (ISV), which excludes an entire study for testing. Whereas RCV hardwires an assumption of training and testing on identically distributed data, this key property is lost in ISV, yielding systematic decreases in performance estimates relative to RCV. Measuring the RCV-ISV difference as a function of number of studies quantifies influence of study-effects on performance.

**Results:**

As a case study, we gathered publicly available gene expression data from 1,470 microarray samples of 6 lung phenotypes from 26 independent experimental studies and 769 RNA-seq samples of 2 lung phenotypes from 4 independent studies. We find that the RCV-ISV performance discrepancy is greater in phenotypes with few studies, and that the ISV performance converges toward RCV performance as data from additional studies are incorporated into classification.

**Conclusions:**

We show that by examining how fast ISV performance approaches RCV as the number of studies is increased, one can estimate when “sufficient” diversity has been achieved for learning a molecular signature likely to translate without significant loss of accuracy to new clinical settings.

## Introduction

There has been substantial effort to develop disease diagnostic strategies based on analyzing large-scale molecular information (i.e., omics data) from patients. Numerous studies aiming at developing such molecular diagnostics have examined omics data, both directly [1], [2], [3], [4], [5] and through meta-analyses [6], [7], [8]. Although many reports have shown high performance estimates for predictive disease classification, identifying molecular signatures that give consistent results across multiple trials remains a challenge [9], [10], [11]. This discrepancy between high reported performance estimates and the relative paucity of robust omics-based tests delivered to the clinic was the subject of a recent in-depth study by the United States Institute of Medicine [12]. While the general issues discussed exist across all omics data platforms, herein we will focus on large repositories of transcriptomics data because of broad availability from many studies, especially those conducted on Affymetrix microarrays (the most abundant source), as well as recent RNA sequencing (RNA-seq) data.

A major factor hindering the consistency of identified disease classifiers and their performances stems from variability in omics data attributed to technical and biological influences that are unrelated to the specific phenotypic differences under study. Gathering gene expression data from different batches-processed at a specific experimental site and time–introduces technical variability, termed “batch-effects” [13]. Moreover, diversity among studies is present and often significant even in the absence of batch-effects because of intrinsic biological variation, including geographic differences in patient subpopulations due to disease heterogeneity [14], [15], [16], [17], [18]. Both batch-effects and intrinsic biological variation introduce site-specific variability that can bias the selection of classifiers by obscuring the phenotype-specific molecular signal.

We use the term *study-effects* herein to describe the joint variability that stems from both technical variation introduced by batch-effects and the biological variation associated with population heterogeneity. Importantly, the presence of these study-effects is not necessarily a reflection of the quality of the laboratories or experimental studies; rather, they emphasize that measured gene expression is sensitive to a broad range of influences. Although numerous excellent studies have examined [19], [20], [21] and attempted to mitigate [22], [23], [24], [25], [26], [27], [28], [29], [30], [31] site-specific variability from technical batch-effects, which have been summarized and compared elsewhere [22], [32], [33], [34], no definitive solution for study-effects has been adopted by the molecular diagnostic community at large.

The motivation of our study is to examine the influence of study-specific variability in gene expression data on disease classification prediction error and suggest how to mitigate this influence to achieve improved classification performance. Our approach to measuring the influence of study-effects on classification involves assessing classification performance with a study-centric validation strategy. In *inter-study validation* (ISV), we identify phenotype-specific classifiers based on data pooled from all studies except for one, and then evaluate the predictive performance on the excluded study. This process is repeated for all studies, leaving each one out for testing and training a predictor on the data combined from all others. This differs from randomized cross-validation (RCV), the standard in machine learning, wherein a random subset of the pooled data, for example ten percent in ten-fold RCV, is set aside for testing, and the predictor is identified from the pooled data excluding this subset. This process is then repeated, for example ten times. The critical difference between these validation strategies is that RCV (and other methods that split data randomly) estimates classification error under a condition (namely random sampling) in which the training and testing data are drawn from the same distribution. In other words, the assumption is made, at least implicitly, that future samples from other studies encountered by the classifier will display the same statistical properties as the training data–a condition that is often violated in real world settings. Hence, randomized sampling obscures systematic differences in expression distributions associated with study-effects. In contrast, ISV is sensitive to study-effects because it preserves the variation among studies (i.e. the training and test sets are not necessarily identically distributed). Consequently, estimates of classifier performance derived using ISV are often low when there are expression patterns that vary substantially between studies within phenotypes. Therefore, the magnitude of discrepancies between performance estimates from ISV and RCV reflects the extent of study-effects. We refer to the comparison of ISV and RCV performance estimates as *comparative cross-validation analysis* (CCVA). CCVA can be applied in subsets of study comparisons in various ways, for example as a measure of how well controlled a multi-site clinical trial is performing in terms of cross-site variation.

In this study, we use comparative cross-validation analysis to gauge the extent to which study-effects confound gene expression-based disease classification performance. We find that including data from more studies improves representation of biological heterogeneity during the disease signature learning process, which mitigates the influence of study-effects. By tracking the difference between the ISV and RCV performance, we measure the extent to which introducing heterogeneity into the training data alleviates the influence of study-effects on disease classification and can thereby estimate when sufficient data has been incorporated to generate classifiers that are robust to study-specific biases.

## Materials and Methods

### Data Preprocessing

For the microarray data, we download the raw.CEL files of each microarray experiment, either from the Gene Expression Omnibus [35], ArrayExpress [36], or from files kindly provided by the original authors of the experiments. We have developed a custom pipeline in MATLAB to preprocess the.CEL files of the samples across all studies in a consensus set using the GCRMA method [37] (see Text S1 for details). We provide this uniformly processed dataset to the community as a resource to download at our website (https://price.systemsbiology.net/measuring-effect-inter-study-variability-estimating-prediction-error), and we have added our uniformly preprocessed dataset to GEO with accession number: GSE60486. It should be noted that this dataset is a compilation and standardization of data generated from other groups.

For the RNA-seq data, we download raw fastq files from the Sequence Read Archive [38] or CGHUB and extracted gene expression counts using STAR alignment [39] and HTSeq software (http://www-huber.embl.de/users/anders/HTSeq/doc/overview.html). We consolidate the data across studies and perform correlation analysis using Python.

### Comparative cross-validation analysis (CCVA)

To estimate the significance of study-effects on classification performance, we compare two metrics for evaluating classification sensitivity: inter-study validation (ISV) and the commonly used randomized cross-validation (RCV). For each method, classification performance metric we use is the phenotype-specific *sensitivity*, defined as the fraction of samples of that phenotype that are correctly identified by the learned molecular signature.

In ISV, the expression data from each experimental study is excluded from the training set used to find diagnostic classifiers. Data from the remaining studies is used to train a classifier using an algorithm of choice. The data from the excluded study acts as a test set to evaluate the performance of a classification algorithm. This ISV is repeated for every study included in the analysis, and the sensitivity for each phenotype evaluated on each excluded study is reported. To mitigate sample number bias in training, we implemented a stratified training loop (see Text S1 for details).

We implement ten-fold RCV, in which one-tenth of the samples combined from all studies for each phenotype is randomly excluded from the training set in each iteration of validation. Classifier training is executed on the remaining nine-tenths of the data, and the excluded tenth of the data is used to evaluate predictive sensitivity. The process is iterated ten times so that each excluded test set is disjoint. The average sensitivity across all ten iterations for each phenotype is reported.

For each phenotype, we compare the average of the ISV sensitivities across all studies of that phenotype against the phenotype-specific sensitivity obtained by ten-fold RCV. This approach is implemented in MATLAB (see https://price.systemsbiology.net/measuring-effect-inter-study-variability-estimating-prediction-error for code).

### Cumulative CCVA

We also investigate the effect of including different numbers of studies in the considered dataset on ISV and RCV outcomes. In this cumulative CCVA, we focus on the three phenotypes with greater than five independent studies: ADC, SCC, and NORM. For each of these phenotypes, we combine data from 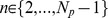 studies of the phenotype under consideration (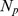 is the total number of studies for the phenotype under consideration) along with data from studies that lack the phenotype under consideration. We perform ISV and RCV on this study-controlled dataset and report the ISV and RCV sensitivities for the phenotype under consideration. For each *n*, five random selections of *n* studies are selected for inclusion, and the ISV and RCV results are averaged over these independent iterations. Note that applying this analysis to a dataset consisting of 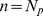 studies of the phenotype under consideration would be equivalent to the standard CCVA. This approach is implemented in MATLAB (see https://price.systemsbiology.net/measuring-effect-inter-study-variability-estimating-prediction-error for code).

### Classification methods applied

We evaluate predictive performance of support vector machine (SVM) [40] and Identification of Structured Signatures And Classifiers (ISSAC) [8]. We choose to use these algorithms because in addition to demonstrating significant classification capability, the methods are based upon disparate classification strategies. SVM was designed to find an optimal discriminating hyperplane based on a set of input feature genes, which we select using the F-score feature selection metric [41] (see Text S1 for details). In contrast, ISSAC was designed to select an optimized set of feature pairs as a multi-phenotype classifier, wherein classification was based on comparing expression values within each pair. We perform feature selection based on the training data within each iteration of validation. We use existing MATLAB implementations of these algorithms [8], [42].

### Statistical significance testing

Non-parametric significance tests are used where possible, with *p*<0.05 set as the significance threshold.

## Results

### Overview of microarray data assembled

We have assembled lung-related expression data from two publicly available online databases: Gene Expression Omnibus [35] and ArrayExpress [36]. Our analysis focuses on the lung because lung diseases pose significant health challenges that would benefit from improved diagnostic methods [5], [43], [44], [45], [46] and because there exists a wealth of gene expression datasets generated repeatedly for multiple lung diseases. We examine data from non-diseased tissue (NORM) and from five common lung disease phenotypes: three types of non-small-cell lung cancers (adenocarcinoma (ADC), squamous cell carcinoma (SCC), and large cell lung carcinoma (LCLC)) as well as two non-cancer diseases (asthma (AST) and chronic obstructive pulmonary disease (COPD)). These studies were performed by laboratories that sampled geographically distinct patient populations; used different protocols for tissue sample collection and preparation; measured gene expression using different Affymetrix microarray platforms; and utilized different data preprocessing methods to yield gene expression values from hybridization intensities measured by the microarrays. We restrict ourselves to microarray platforms developed by Affymetrix to eliminate additional sources of variability associated with microarray technology, which have been covered elsewhere in the literature [15]. Our assembled gene expression dataset consists of 1,470 samples collected by 26 independent experimental studies. A summary of the data included in our analysis is shown in Table 1 (see Table S1 for a complete list of experimental study sources; some studies measured more than one phenotype considered in our analysis). Microarray sample sizes within each phenotype ranged from 49 to 580. In each case, we preprocess the raw data using our custom pipeline to create a consistent dataset with minimal algorithmic sources of variance (see Text S1 for rationale and details). We use this uniformly preprocessed dataset for all subsequent classification analyses described in this study.

**Table 1:** Summary of lung disease microarray data.

Disease	Label	Platforms	# Studies	# Samples	Sampling Method
**Adenocarcinoma**	ADC	1,2	14	580	A
**Squamous Cell Carcinoma**	SCC	1,2	7	239	A
**Large Cell Lung Carcinoma**	LCLC	1,2	4	49	A
**Asthma**	AST	1	3	70	B,C
**Chronic Obstructive Pulmonary Disease**	COPD	1,2	4	63	A,B
**Normal**	NORM	1,2	17	469	A,B

The number of samples (n = 1470), number of studies (n = 26), types of platforms, and the methods of tissue extraction used to collect samples in the studies are shown. The platform labels represent: 1) Affymetrix Human Genome U133 Plus 2, and 2) Affymetrix Human Genome U133A. The sampling method labels represent: A) surgical resection, B) bronchoscopy brushing, C) bronchoalveolar lavage. See Table S2 for detailed information on the studies.

### Comparative cross-validation analysis evaluates influence of study-effects on classification performance

**Figure 1:**
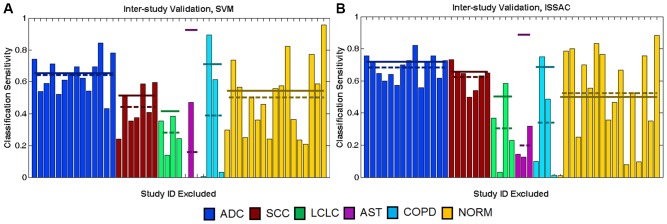
Inter-study validation and randomized cross-validation performance. The graphs show ISV and RCV results from SVM (A) and ISSAC (B). For clarity, the Study ID labels have been excluded from this visualization (see Text S1 for expanded versions of these plots that include the individual Study ID labels). The colored bars report sensitivities achieved on the validation study designated in the horizontal axis (e.g., the bar on the farthest left in (A) shows that 74% of ADC samples in the first ADC study are correctly classified by SVM when that study is excluded from training). The order of studies in the horizontal axis is identical for panels (A) and (B). Dashed lines represent average ISV sensitivities for each phenotype. Solid lines report corresponding ten-fold RCV sensitivities of each phenotype.

We apply CCVA on our lung microarray dataset using two very different multi-class classification schemes–the commonly used linear one-versus-one multiclass support vector machines (SVM) [40] and Identification of Structured Signatures And Classifiers (ISSAC) [8] (see Text S1 for descriptions of these algorithms)–to demonstrate that the results throughout are largely independent of the classification method selected. Figure 1 shows the estimated ISV sensitivities for each study, grouped by phenotype, calculated by ISSAC and SVM. The figure also shows the average ISV sensitivities across all studies of each phenotype (dashed lines), as well as the sensitivities obtained by ten-fold RCV for each phenotype (solid lines). The qualitative outcomes of ISV were consistent across the two classification schemes. The consistency of the results provides evidence that results herein are largely independent of the specific classification method used. There was no significant correlation between ISV or RCV performance with sample size of study and no significant correlation between ISV performance and RCV performance (p>0.05, see Text S1 for details and for plot of ISV performance as function of study sample sizes).

### Number of independent studies affects the influence of study-effects on performance

The results in Figure 1 show that phenotypes with data from larger numbers of studies have smaller differences in RCV and ISV performance because their ISV sensitivities are greater than those in phenotypes with fewer studies. We observe a significant negative correlation between the difference of ISV and RCV sensitivity across all studies for a phenotype and the number of studies belonging to that phenotype (Spearman’s rho = −0.93, p<0.05 for SVM and ISSAC). ISV sensitivities of SCC, ADC, and NORM–phenotypes with data from seven or more independent studies-differ from RCV sensitivities only by 0.07, 0.01, and 0.04, respectively for SVM, and by 0.03, 0.03, and 0.02 for ISSAC. In contrast, phenotypes with data from few studies independent studies (AST, 3 studies; LCLC, 4 studies; COPD, 4 studies) have low ISV sensitivities even when the corresponding sensitivities from RCV are high, resulting in greater gaps between RCV and ISV performance (RCV - ISV of AST = 0.76, LCLC = 0.13, and COPD = 0.32 for SVM; RCV- ISV of AST = 0.69, LCLC = 0.20, and COPD = 0.35 for ISSAC).

Besides yielding improved ISV performance relative to RCV, phenotypes with more studies achieve more consistent ISV results. We quantify ISV consistency by the coefficient of variation (CoVar), which is equal to the standard deviation of ISV sensitivities across studies belonging to a particular phenotype divided by the average. Lower CoVar values translate to higher consistency. Phenotypes with fewer studies, such as AST and COPD, have low consistency (CoVar_AST_ = 1.7, CoVar_COPD_ = 1.1 for SVM; CoVar_AST_ = 0.54, CoVar_COPD_ = 1.0 for ISSAC), whereas SCC and ADC have higher consistency (CoVar_SCC_ = 0.30, CoVar_ADC_ = 0.17 for SVM; CoVar_SCC_ = 0.12, CoVar_ADC_ = 0.11 for ISSAC).

**Figure 2:**
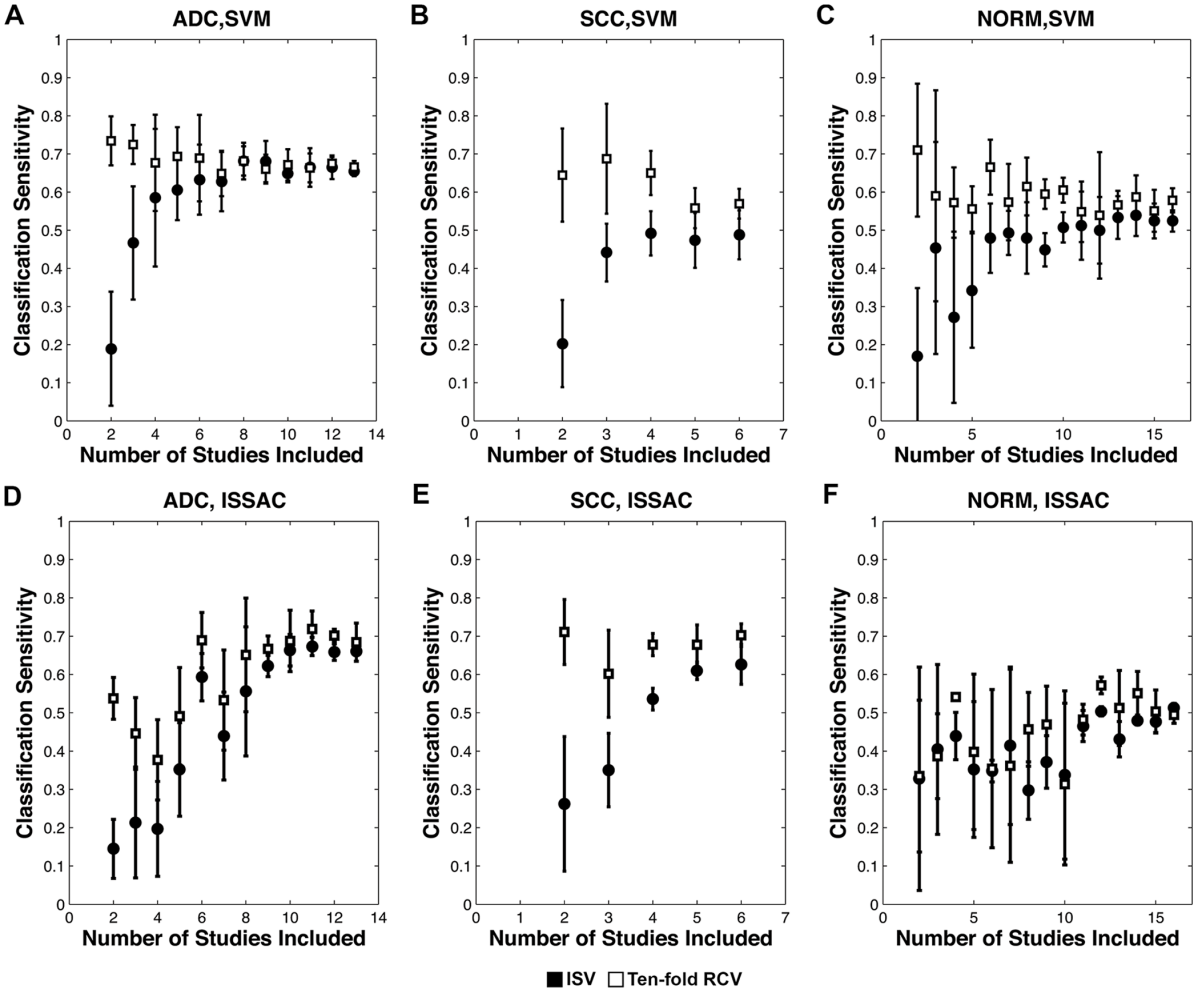
Inter-study-validation and randomized cross-validation results as function of number of studies included in analysis. Average ISV (black circles) and RCV (white squares) sensitivities as a function of the number of studies included, for ADC (A, D), SCC (B, E), and NORM (C, F), using SVM and ISSAC classifiers.

To measure the extent to which the number of studies affects CCVA performance, we calculate ISV and RCV while varying the number of studies included in each phenotype. Figure 2 shows the phenotype-averaged ISV and RCV sensitivities for ADC, SCC, and NORM, as the number of studies considered in CCVA is varied based on SVM and ISSAC (see Text S1 for plot of ISV sensitivities as a function of the training set sample sizes). Each point in the figure represents the average and standard deviation of ISV and RCV estimates calculated from five independent sampling combinations of input training studies (see Cumulative CCVA section in Methods for details).

When only two studies are considered in analysis, average ISV sensitivity for each phenotype is substantially lower than the corresponding RCV sensitivity, showing that results learned from one study context do not translate well to a second. Each phenotype achieves higher sensitivity when data from a greater number of studies are used for signature learning (Spearman’s rho = 0.65 for SVM, rho = 0.57 for ISSAC; p<0.05 for both methods correlating mean ISV sensitivity with the number of phenotype-specific studies included over all three phenotypes). Moreover, we observe a converging of ISV sensitivity toward the corresponding RCV sensitivity as additional studies are added, although the rates of convergence differ between phenotypes. As the ISV sensitivity approaches RCV sensitivity, the incremental improvement of average ISV sensitivity drops with the addition of further studies. We also observe a significant negative correlation between the CoVar and the corresponding ISV sensitivity (Spearman’s rho = −0.64 for SVM, rho = −0.78 for ISSAC; *p*<0.05 for both methods correlating over all three phenotypes), indicating enhanced signature performance and consistency with the addition of data from different studies. Further analysis shows that the trends of ISV performance are reflected in large-scale differences in the expression profiles that can be visualized by principal component analysis, and that these performance trends also have association with consistency of selected gene signatures (see Text S1). These results demonstrate that integrating gene expression data across diverse studies can strengthen phenotype-associated signal that translates into new study contexts.

### Study-effects similarly impact classification performance in RNA-seq data

Advances in sequencing technologies have recently enabled large-scale RNA-seq studies to measure gene expression for disease classification [47]. Although RNA-seq offers many advantages over microarrays [47], [48], study-specific variability has also been observed in RNA-seq data [20], [21]. To evaluate the extent that the CCVA results from microarray data also apply to RNA-seq, we examined RNA-seq data collected from four independent studies consisting of ADC and NORM data: GSE37764 (11 ADC, 12 NORM samples) [49], ERP001058 (90 ADC samples and 76 NORM samples) [50], TCGA (448 ADC samples) [51], and dbGaP (132 NORM samples) [52]. Correlation and classification analysis based on study label on data from GSE37764 and ERP001058 confirm that study-effects are also influencing these RNA-seq datasets (see Text S1).

**Figure 3:**
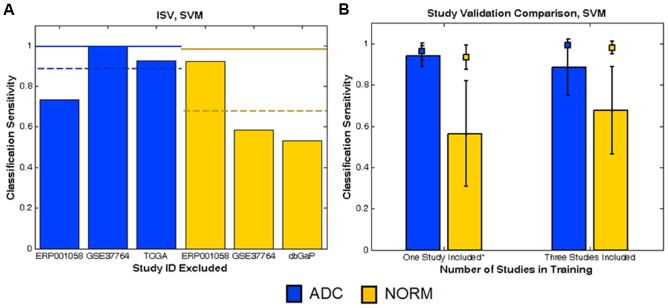
Inter-study validation performance in RNA-seq data based on SVM. (A) The colored bars report ISV sensitivities achieved by validating performance on the study designated in the horizontal axis. Dashed lines represent average ISV sensitivities for each phenotype. Solid lines report corresponding ten-fold RCV sensitivities of each phenotype. (B) The colored bars report average sensitivities from validating on studies excluded from training. Squares represent corresponding RCV sensitivities from the studies included in the training set. Results were averaged across the different combinations of training studies, and the error bars report the standard deviation of the results.

The CCVA results on data from these four studies indicate two key results. First, the gap between RCV and ISV performance is smaller for ADC when the RNA-seq studies are analyzed (RCV-ISV difference of 0.10, Figure 3A) than when the same number of ADC microarray studies is analyzed (difference of 0.26). This suggests that ADC classification based on RNA-seq data from three independent studies is not significantly impacted by study-effects (an improvement over ADC classification based on the same number of microarray studies). To substantiate this result, we further estimated classification performance when only one study was used to train a classifier (Figure 3B). The bars represent classification sensitivities achieved on the studies excluded from training, whereas the square points represent RCV sensitivities on the training studies. ADC sensitivity on studies excluded from training (0.94±0.05) remains high and close to the RCV sensitivity (0.96±0.04) even when only one study is used to train.

Second, we find that, as with microarray data, increasing RNA-seq dataset diversity by including additional studies into the analysis also mitigates the impact of study-effects on classification outcomes. For example, ISV sensitivity calculated from NORM RNA-seq data (0.68±0.21) is significantly lower than corresponding RCV sensitivity (0.98±0.03) (p<0.05, Wilcoxon ranksum test), indicating that NORM sensitivity is indeed compromised by study-effects. Notably, we find that NORM ISV sensitivity, which is achieved by learning a molecular signature from two NORM training studies (0.68±0.21), is greater than the sensitivity achieved when only one NORM study was used to learn a molecular signature (0.56±0.26). Increasing the number of RNA-seq studies included in training improves NORM classification performance on untrained studies and does not significantly change ADC performance on untrained studies, suggesting that greater benefit in classification can be gained by the integration of data from additional NORM RNA-seq studies.

## Discussion

Generating molecular signatures that yield consistently high predictive capability in diagnostic tests remains a critical challenge in omics-based biomarker discovery. We find that study-effects stemming from both technical and biological variability substantially decrease predictive performance and consistency when data from only a few studies are considered in learning molecular signatures. However, our results show that incorporating data from additional independent studies mitigates the impact of study-effects, thereby reducing the predictive error. The qualitative trends of our study-effects results remained consistent across microarray and RNA-seq datasets. Given that study-effects also account for intrinsic biological heterogeneity, the trends associated with study-effects are relevant to classification even with improved technologies.

A significant source of variability arises not from technical batch-effects but rather is an inevitable consequence of the inherent heterogeneity of many disease phenotypes. Given that study-effects associated with disease heterogeneity are biological, they can best be accommodated by collecting data from multiple sources. The key point is that the diversity represented in the training and test set needs to reflect the range of diversity expected in the clinical setting. Subpopulations from different studies have different underlying distributions of expression, so sampling data from multiple independent studies improves the approximation of the global distribution across multiple sites – even when sample sizes are held constant – which aids the identification of consistent classifiers. Therefore, the improvement in classification performance that results from training classifiers on larger numbers of studies highlights the need to incorporate more population heterogeneity in future biomarker discovery studies by integrating data from multiple sites, including additional sites in test validation.

Comparative cross-validation analysis provides a quantitative basis for prioritizing strategies for improving classification of different phenotypes. For example, our CCVA results highlight phenotypes for which diagnostic reproducibility was most greatly affected by study-effects. These phenotypes are the most suitable candidates for further data gathering and analysis to immediately yield better classification outcomes. In contrast, in phenotypes with average ISV sensitivities that approach RCV sensitivities, our results suggest that, because of the difficulty of the gene expression-based classification problem, simply gathering more gene expression data and using the same algorithms would not likely substantially improve performance. In these cases, leveraging other strategies for classification, including redefinitions of molecular phenotypes, integration of multi-omic data, and contextualization with biological networks, may be more beneficial to finding classifiers with more consistent performance. By measuring the improvement in classification once study-effects have been mitigated, CCVA can be used to guide future data gathering efforts.

## Conclusions

In this study, we quantify the degree of impact of technical and biological “study-effects” on disease classification performance. We find that learning diagnostic signatures on larger numbers of studies compensates for study-effects and results in marked improvements in classification performance. Moreover, we can estimate when “sufficient” diversity has been achieved for learning classifiers that are likely to translate effectively to new clinical settings. These results are relevant to phenotype prediction using data across measurement technologies. Our finding that study-effects can be quantifiably mitigated by introducing data collected from additional studies has applicability to disease classification study design strategies. It has clear implications for study design because diversity of samples in the training set (e.g. from multiple sites) shows markedly better consistency in predictive accuracy when taken to new clinical sites, as is a needed step on the path to clinical use. This underscores the need to incorporate more population heterogeneity in future classification studies by integrating data from multiple sources. Additionally, we find that different phenotypes require different degrees of training heterogeneity to mitigate study-effects. Applying comparative cross-validation analysis, we can discriminate between phenotypes that would benefit from for further data gathering to increase training heterogeneity from the phenotypes may require different analysis strategies to reduce predictive error. Therefore, our approach provides a computational tool for prioritizing strategies to improve disease classification.

## Supporting Information

Table S1 **Microarray Data Details.** This contains detailed information of each of the experimental studies that had data included in our analysis.
(XLSX)

Text S1 **Supplemental Document**. This contains descriptions of supplementary analyses and descriptions of methods.
(DOCX)
