# Supplementary material for: Measuring the Effect of Inter-Study Variability on Estimating Prediction Error
Source: PLoS One. 2014 Oct 17;9(10):e110840. doi: 10.1371/journal.pone.0110840 (PMC4201588; doi:10.1371/journal.pone.0110840)
Supplement: Text S1 — Supplemental Document. This contains descriptions of supplementary analyses and descriptions of methods. (DOCX) [file pone.0110840.s002.docx]

SUPPLEMENTAL INFORMATION

**Measuring the effect of inter-study variability on estimating prediction error**

Table of Contents

[Technical batch-effects can arise from differences in microarray preprocessing 2](#_Toc397094986)

[Mitigating batch-effect with consensus preprocessing 3](#_Toc397094987)

[MAS5call filtering 3](#_Toc397094988)

[Study effects are observed in RNA-seq data 4](#_Toc397094989)

[Extended comparative cross-validation analysis (CCVA) results 6](#_Toc397094990)

[Correlation between inter-study validation, randomized cross-validation, and sample size 6](#_Toc397094991)

[Extended cumulative comparative cross-validation analysis results 8](#_Toc397094992)

[Varying numbers of ADC studies 9](#_Toc397094993)

[Varying numbers of SCC studies 10](#_Toc397094994)

[Varying numbers of NORM studies 10](#_Toc397094995)

[Extended principal component analysis results 11](#_Toc397094996)

[Impact of study-effects on the consistency of gene classifiers 15](#_Toc397094997)

[Description of disease classification algorithms used 15](#_Toc397094998)

[Support Vector Machines 15](#_Toc397094999)

[Identification of Structured Signatures And Classifiers 18](#_Toc397095000)

[Training set selection in validation 19](#_Toc397095001)

[References 19](#_Toc397095002)

# Technical batch-effects can arise from differences in microarray preprocessing

A significant source of technical batch-effects comes from the data preprocessing required to yield gene expression values from microarrays. The raw Affymetrix oligonucleotide microarray measurements require *in silico* preprocessing to reconcile them into expression values. Several different preprocessing methods have been developed to perform this analysis; the three methods used by the studies included in our meta-analysis were MAS5 [1], RMA [2], and GCRMA [3].

We study the effect of including data processed with different procedures on binary TSP and SVM classification performance on a small test subset of the meta-analysis data. The subset incorporated data from ADC (77 samples from GSE10245, GSE17475, and GSE10799) and SCC (75 samples from GSE10245, GSE2109, and GSE6253). The raw intensity files were compiled and each sample was preprocessed with both RMA and GCRMA using MATLAB. We assemble datasets composed of different mixtures of preprocessing methods (i.e.: 0%, 25%, 50%, 75%, 100% RMA, remainder GCRMA) and compare the classification accuracies achieved. For each input dataset containing both RMA and GCRMA preprocessed data, we test 20 dataset splicing permutations that preserved the RMA/GCRMA ratio.

We find that binary classification accuracy is significantly reduced when we include datasets generated by heterogeneous preprocessing methods (p < 0.05 Wilcoxon ranksum test) (see Figure 1A). We further find that the TSP algorithm tends to select the same classifiers consistently across different rounds of randomized cross validation when the consistent preprocessing had been applied to all samples, whereas the algorithm tends to select disjoint classifiers during different rounds of cross validation when a mix of the preprocessing schemes were applied to the samples (see Figure 1B).


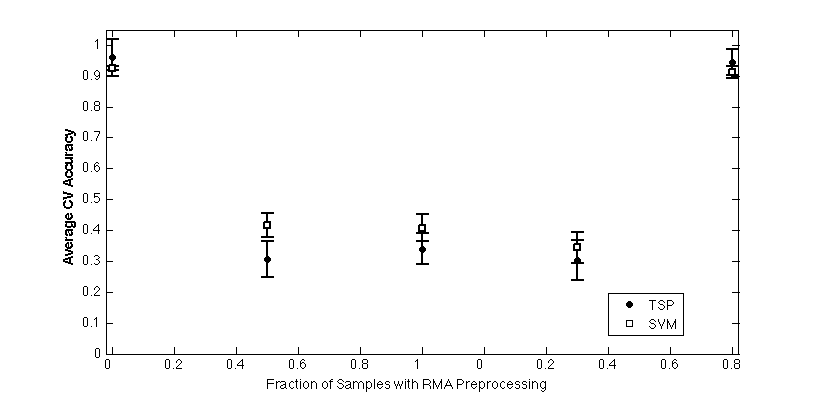
**
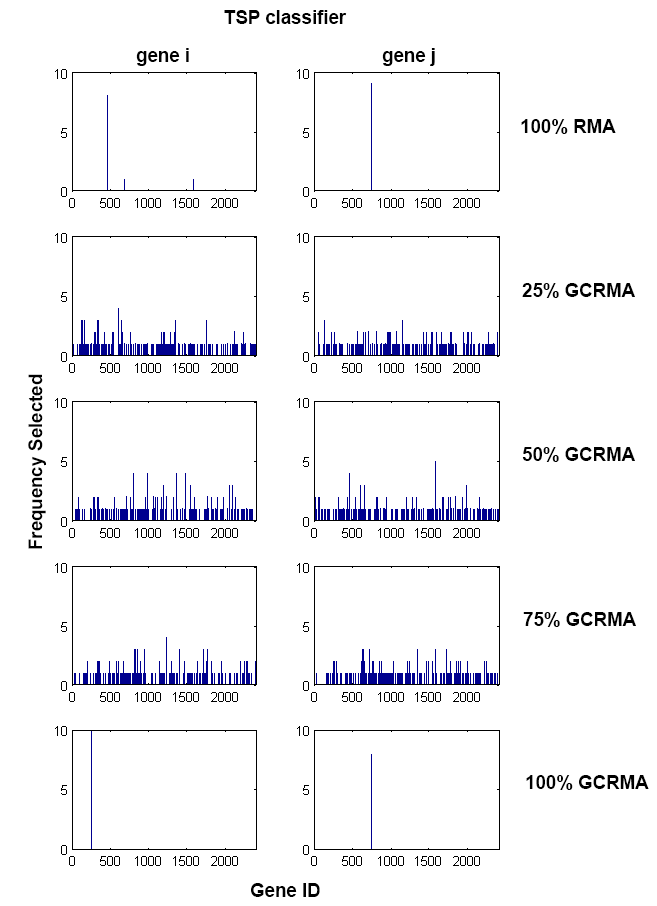
**

A

B

**Figure 1. Performance of binary TSP and SVM classification at different RMA/GCRMA preprocessing ratios**. (A) Accuracy of classifying between ADC and SCC. The x-axis indicates the percent of data included in training that were processed by RMA. The remaining fraction of the data were processed by GCRMA (1-%RMA samples). The error bars represent standard deviation from 20 permutations. (B) Consistency of TSP classifiers selected among different iterations of cross-validation for different fractions of samples with RMA processing. The x-axis show the gene ID number, and the y-axis indicates the frequency that a particular gene was selected as a TSP classifier in an iteration of cross-validation.

Datasets with mixed RMA/GCRMA processed samples show that different genes were selected as classifiers in different iterations of cross-validation. In contrast, datasets with only RMA or only GCRMA processing yield classifiers that are repeatedly selected across different iterations of cross-validation. This suggests that RMA and GCRMA transformed the data in disparate ways, so that mixing preprocessing procedures introduces a source of non-phenotype associated variability. To mitigate the variability introduced to the data by mixed preprocessing, we preprocess the raw .CEL files of all of our samples in a consensus set with the approach described below, and we use this consensus-preprocessed dataset for all of the classification analyses described in this paper.

## Mitigating batch-effect with consensus preprocessing

The Affymetrix oligonucleotide microarrays measure gene expression by quantifying intensity of fluorescently labeled gene fragments that bind to sets of 25-mer oligonucleotide probes on the chip with specific sequences tailored to be complementary to the target genes. In addition to ‘perfect match’ probes that are perfectly complementary to regions of a target mRNA, Affymetrix microarrays also have ‘mismatch’ probes, which contain a mismatched nucleotide halfway along the probe sequence (position 13). These mismatch probes attempt to estimate non-specific binding for the probes corresponding to each mRNA gene sequence. Each gene is generally associated with a ‘probe set’ consisting of several pairs (usually 11) of perfect match and mismatch probes. The raw measurements for each probe set consist of a set of intensities from the probes, which require *in silico* preprocessing by (1) correcting for background variability, (2) normalizing intensities across samples, (3) and summarizing intensities across the probe set into a single expression value. The output of the summarization step corresponds to the background adjusted expression value for the mRNA of interest.

We preprocess using the MATLAB implementation of GCRMA, which corrects for background variability by accounting for optical noise, probe affinity, and ‘mismatch’ probe adjustment; normalizes intensities by quantile normalization; and summarizes intensities using a median polish method [3]. To minimize preprocessing batch effects, it is desirable to preprocess all samples in the entire dataset together. Unfortunately, preprocessing requires microarray platform-specific chip specifications indicating the locations of each probe on the chip, precluding global preprocessing on meta-analyses that use multiple platforms. To address this problem, we have developed software to combine the raw CEL data files from multiple platforms that share the same probe sets. Importantly, probe sets across the platforms we consider contain the same probe sequences. These “consensus datasets” are then preprocessed normally using GCRMA. The output of this consensus preprocessing contains only the 22,277 probes that exist on both microarray platforms that we use; probes that only appear on one of the platforms are excluded from preprocessing and subsequent consideration.

## MAS5call filtering

The Affymetrix oligonucleotide microarrays standard data preprocessing software, MAS5, includes a function that assesses the expression value of each gene as ‘present,’ ‘marginal’ or ‘absent’ based on whether or not the measured expression of the ‘perfect match’ probes are significantly greater than the ‘mismatch’ probes based on the nonparametric Wilcoxon signed rank test [4]. The ‘present’ call is made when the p-value of difference is less than 0.04, the ‘marginal’ call is made when the p-value of the difference is between 0.04 and 0.06, and the ‘absent’ call is made when the p-value of the difference is greater than 0.06, representing no significant difference between the measurements of the ‘perfect match’ and the ‘mismatch’ probes. These expression quality calls are used as a basis for filtering probes to be considered in the classification scheme.

We have investigated the classification performance effect of excluding expression probes based on varying the threshold of ‘absent’ calls present in the samples. Although the number of genes excluded changes substantially depending on the fractional threshold of ‘absent’ calls that we deem acceptable for a gene to be considered in subsequent classification (ranges from 5818 probes with ‘0% absent’ threshold to 21647 probes with ‘not all samples absent’ threshold), the performance of the TSP tree classification scheme does not significantly change as a function of the ‘absent’ call threshold. We choose to use a 90% absent exclusion threshold; i.e., genes were excluded from consideration if more than 90% of the samples were labeled as ‘absent.’ We perform the MAS5call filtering within the validation loop, excluding genes based on the calls of the training data during each iteration of the validation.

# Study effects are observed in RNA-seq data

Recent advances in sequencing technologies has enabled the use of RNA-seq to measure gene expression [5]. Although RNA-seq offers several advantages over microarrays [5, 6], study-specific variability has been shown to exist in RNA-seq data [7]. We wish to examine whether these study-effects have an impact on phenotype classification performance.

As a test case, we examine RNA-seq data collected from two independent studies that collected both ADC and NORM: GSE37764 [8] and ERP001058 [9], as well as with ADC data from the Cancer Genome Atlas (TCGA) [10] and NORM data from dbGaP [11]. We download raw .fastq files from the Sequence Read Archive [12] or CGHUB and extracted gene expression counts using STAR alignment [13] and HTSeq software (<http://www-huber.embl.de/users/anders/HTSeq/doc/overview.html>).

We first examine the data from the studies that gathered data from both ADC and NORM. Pairwise correlation analysis from the GSE37764 and ERP001058 datasets show that the Pearson correlation coefficients for samples of different phenotypes within a study (average correlation: 0.96) are slightly but significantly higher than the coefficients for samples of the same phenotype from different studies (average correlation 0.95) (p < 0.05). Moreover, when we perform ten iterations of binary classification under ten-fold randomized cross validation (RCV) to distinguish either between phenotype or between studies, we find that classification sensitivity and specificity were significantly higher for predicting study labels than phenotype labels when SVM was used as the classification algorithm (p < 0.05, Wilcoxon ranksum test see Table 1).

**Table 1. Confusion matrices showing classification performance on RNA-seq data based on study labels or phenotype labels.**

| **SVM** |  | **Predicted Study Labels** | |  |  | **Predicted Phenotype Labels** | |
| --- | --- | --- | --- | --- | --- | --- | --- |
|  |  | ERP001058 | GSE37764 |  |  | ADC | NORM |
| **Actual Study Labels** | ERP001058 | 0.994±0.02 | 0.006±0.02 | **Actual Phenotype Labels** | ADC | 0.95±0.07 | 0.05±0.07 |
|  | GSE37764 | 0±0.07 | 0.9933±0.07 |  | NORM | 0.06±0.09 | 0.94±0.09 |

We then integrate the data from GSE37764 and ERP001058 with ADC data from the TCGA and NORM data from dbGaP to test the extent to which classifiers trained from a single study can correctly predict samples collected from other studies. Figure 2 summarizes the sensitivities of classifying ADC (blue bars) and NORM samples (yellow bars) after training SVM classifiers on data only from ERP001058, from GSE37764, or from TCGA and dbGaP (we used TCGA and dbGaP together to train a set of SVM classifiers because each study had only one phenotype). The bars associated with the “CV” categories report the ten-fold RCV sensitivities yielded by testing on samples from the same study as the training study. The grouped bars in the middle and right of each plot represent the independent validation sensitivities from validating on studies not used to train the classifiers. While ADC sensitivity remains high during both randomized cross-validation and validation on independent studies, NORM sensitivity was appreciably lower in validation on independent studies. Collectively, these results suggest that study-effects in RNA-seq data have an impact on phenotype classification.


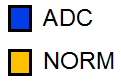

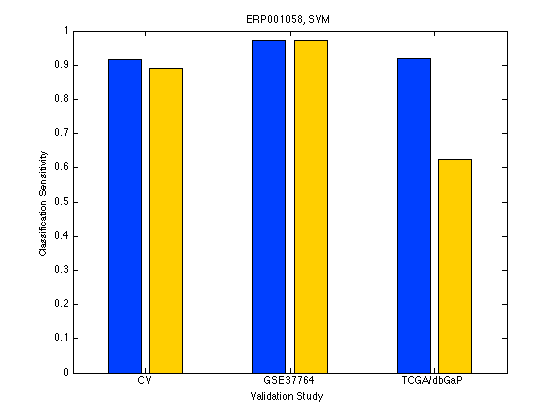

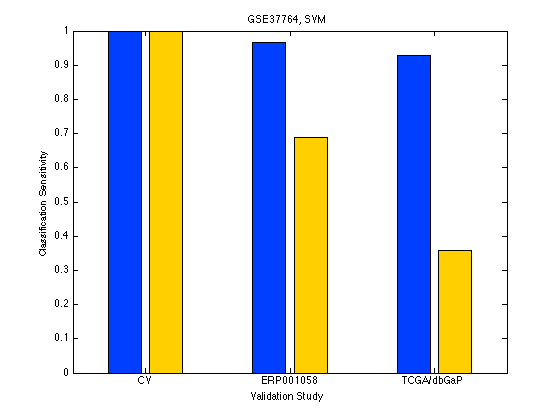

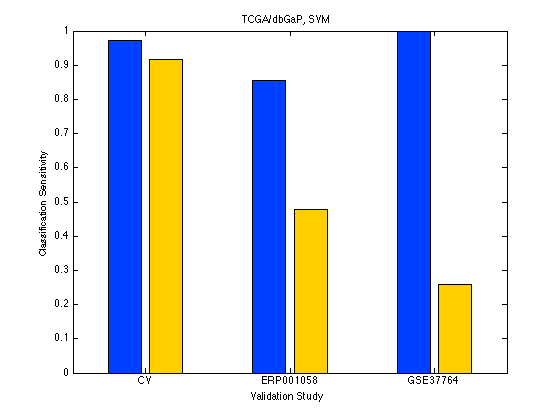
**Figure 2. Classification sensitivities yielded from training on one study.** CV (left-most grouping of bars in each plot) indicates cross-validation sensitivities for ADC (blue bars) and NORM (yellow bars). The other groups indicate the sensitivities yielded from testing on the indicated study or studies. TCGA and dbGaP data were used together to train and test.

# Extended comparative cross-validation analysis (CCVA) results


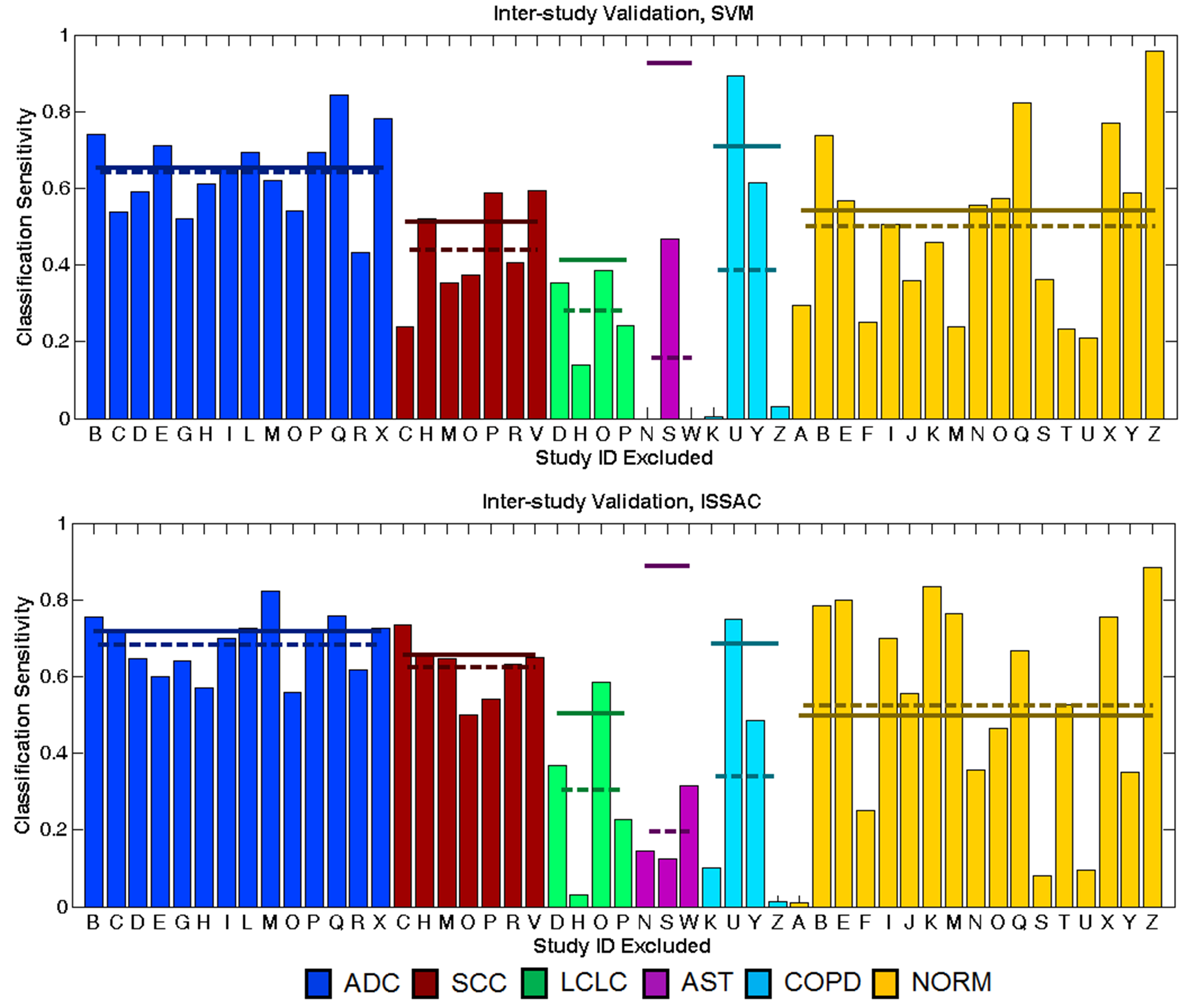


Figure 3. Expanded inter-study validation results. These plots are the same as the graphs featured in Figure 1, with the added Study ID labels (e.g., the bar on the farthest left in (A) shows that 74% of ADC samples in Study B are correctly classified by SVM when Study B is excluded from training). Some Study ID labels appear more than once (e.g. Study H matches both a blue ADC bar and a red SCC bar) because those studies have data from multiple phenotypes, which have sensitivities that are reported separately. The order of studies in the horizontal axis is identical for the two panels.

## Correlation between inter-study validation, randomized cross-validation, and sample size

Figure 4 represents the inter-study validation performance shown in Figure 1 of the main text as a function of the sample sizes of the studies being excluded in each iteration of validation. Figure 5 represents the cumulative comparative cross-validation analysis (CCVA) performance shown in Figure 2 of the main text as a function of the training set sizes in each iteration of validation.


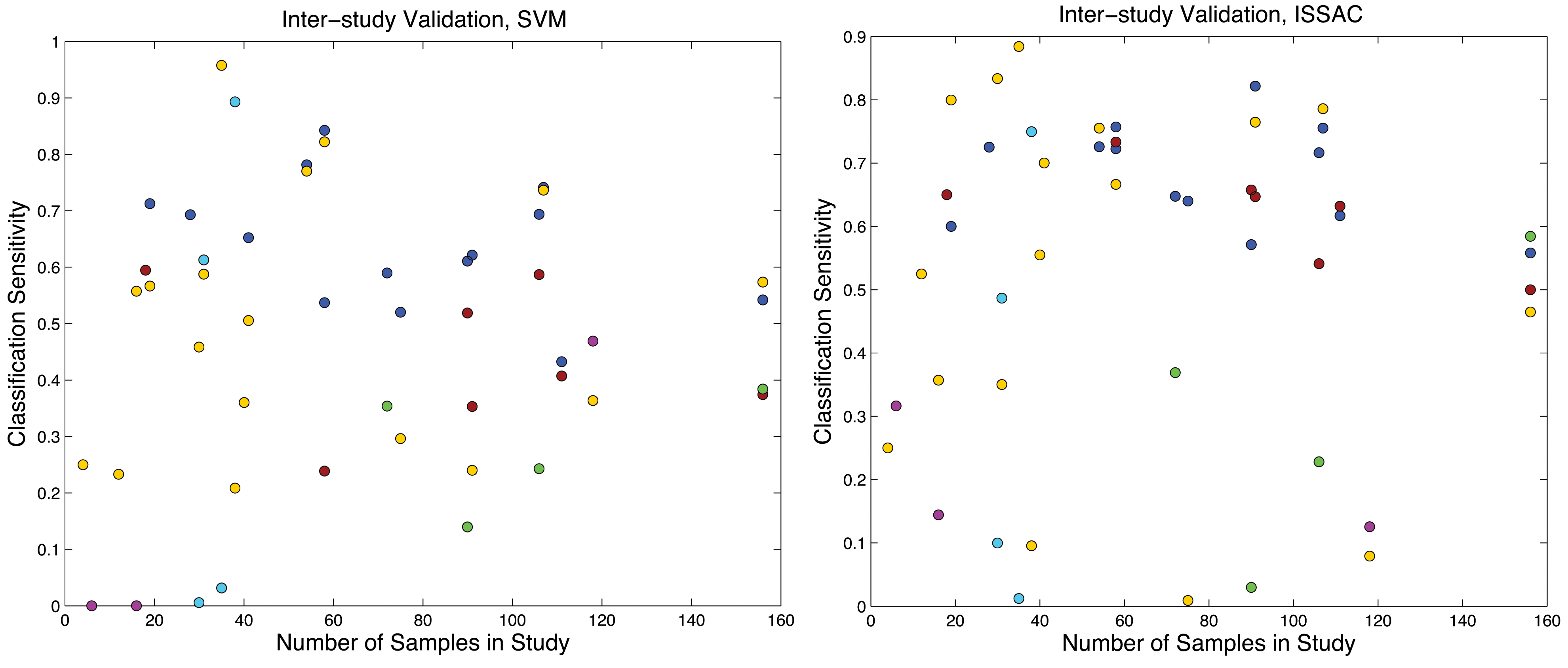


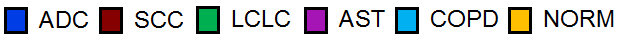


Figure 4. Inter-study validation phenotype-specific sensitivity as a function of study sample size. The points are color-coded according to the phenotype.


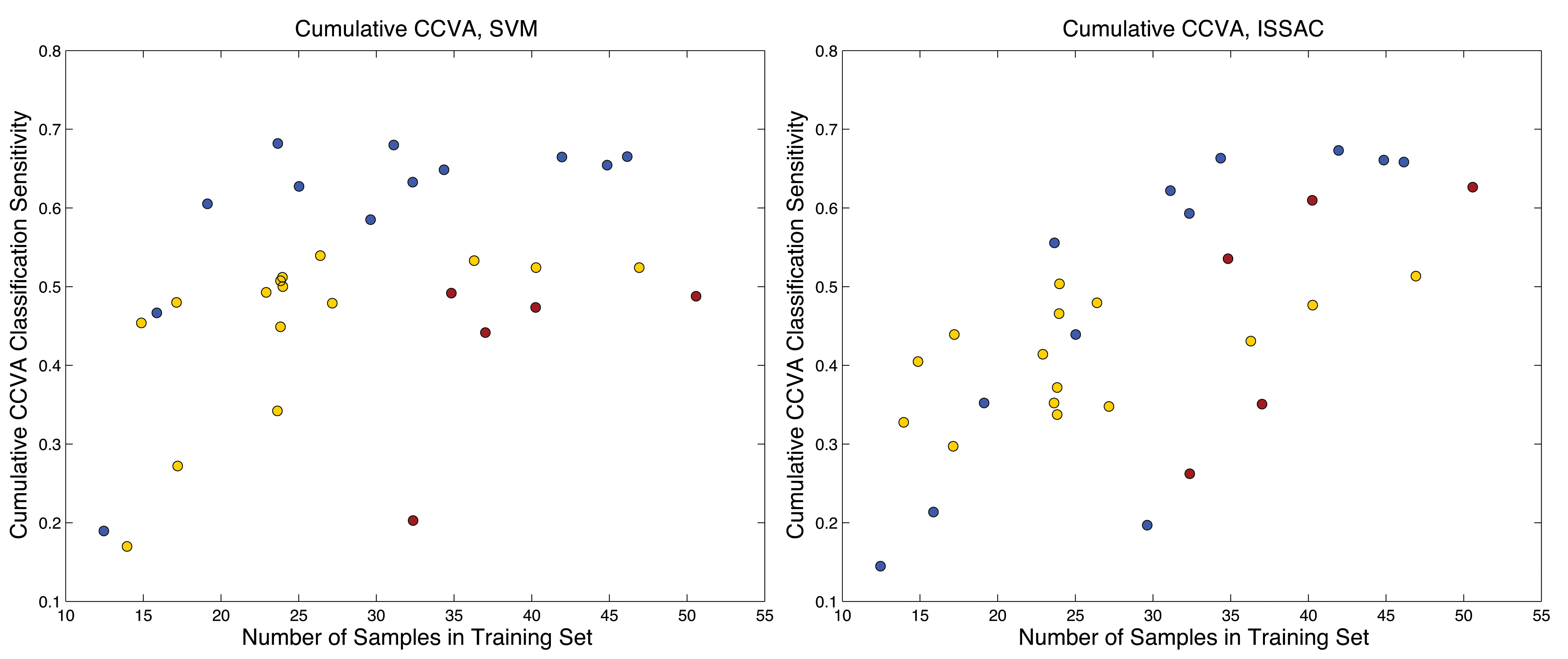


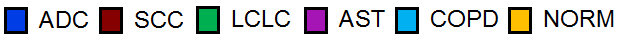


Figure 5. Cumulative comparative cross-validation analysis phenotype-specific sensitivity as a function of training sample size. The points are color-coded according to the phenotype.

We tested whether the trends observed in comparing inter-study validation (ISV) with randomized cross-validation (RCV) could be correlated with sample size of the associated study or phenotype. Figure 6 shows the plot of ISV sensitivity for each study as a function of the study sample size and the plot of RCV sensitivity for each phenotype as a function of the phenotype sensitivity. Correlation analysis revealed that neither ISV nor RCV performance correlated significantly with sample size (Spearman correlation coefficient = 0.2 for ISV vs. study sample size; Spearman correlation coefficient = 0.1 for RCV vs. phenotype sample size; p > 0.05 for both). This suggests that the comparative cross-validation results are primarily associated with factors other than sample size.

Figure 6. Correlation between ISV, RCV and sample number.

Correlation analysis also showed no significant correlation phenotype-averaged ISV sensitivity and RCV sensitivity (see Figure 7, Spearman correlation coefficient = -0.2, p > 0.05). Taking RCV to be a metric of the difficulty of the classification problem, the lack of significant correlation to ISV implies that ISV performance is primarily associated with factors other than the difficulty of the classification problem.

Figure 7. Correlation between average ISV sensitivity and RCV sensitivity.

## Extended cumulative comparative cross-validation analysis results

Figures 8-13 show the extended results of CCVA as a function of the number of studies included in analysis. The figures include analogous results to Figure 2 A-C in the main text that were generated using SVM and ISSAC as the classification algorithm.

## Varying numbers of ADC studies


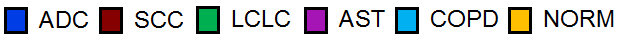


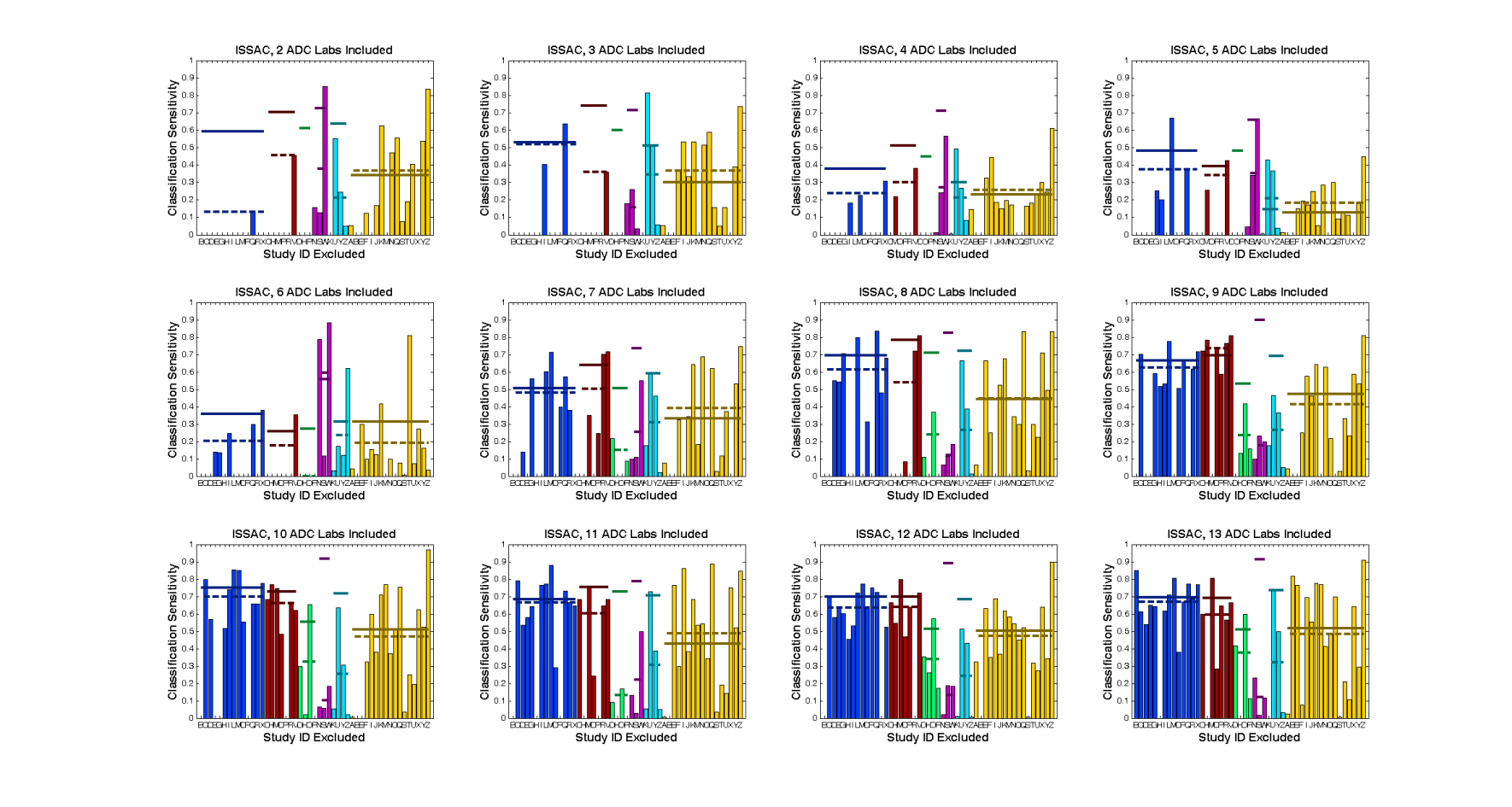


**Figure 8. Inter-study validation results varying ADC studies calculated by SVM.**


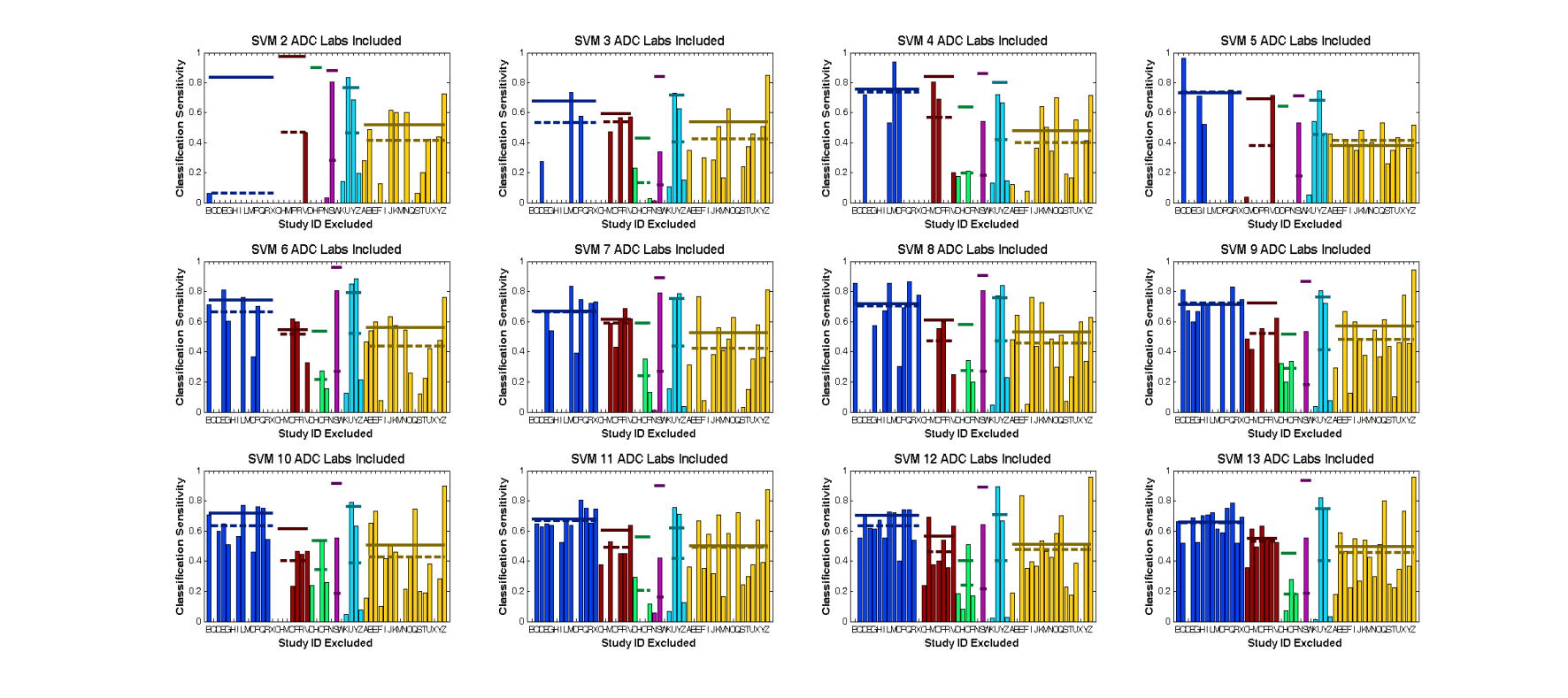


**Figure 9. Inter-study validation results varying ADC studies calculated by ISSAC.**

## Varying numbers of SCC studies


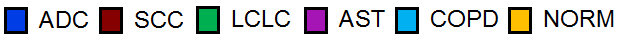


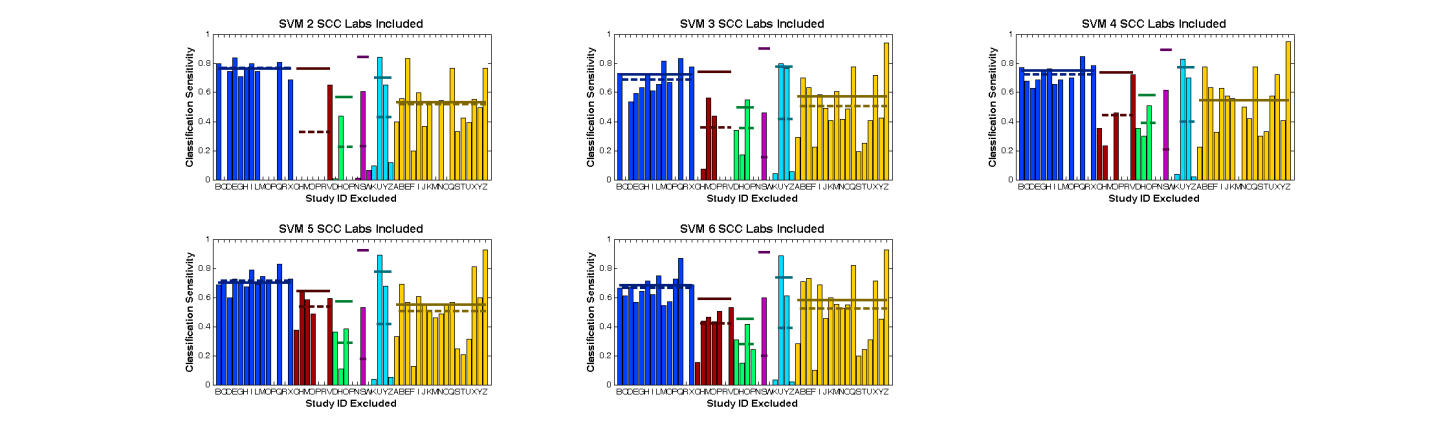


**Figure 10. Inter-study validation results varying SCC studies calculated by SVM.**


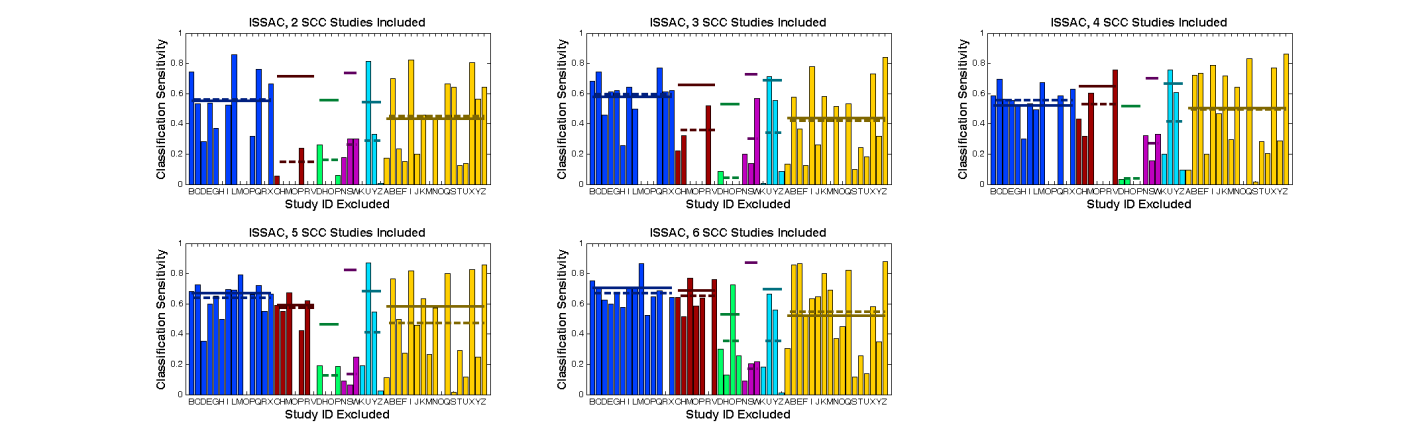


**Figure 11. Inter-study validation results varying SCC studies calculated by ISSAC.**

## Varying numbers of NORM studies


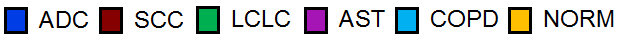


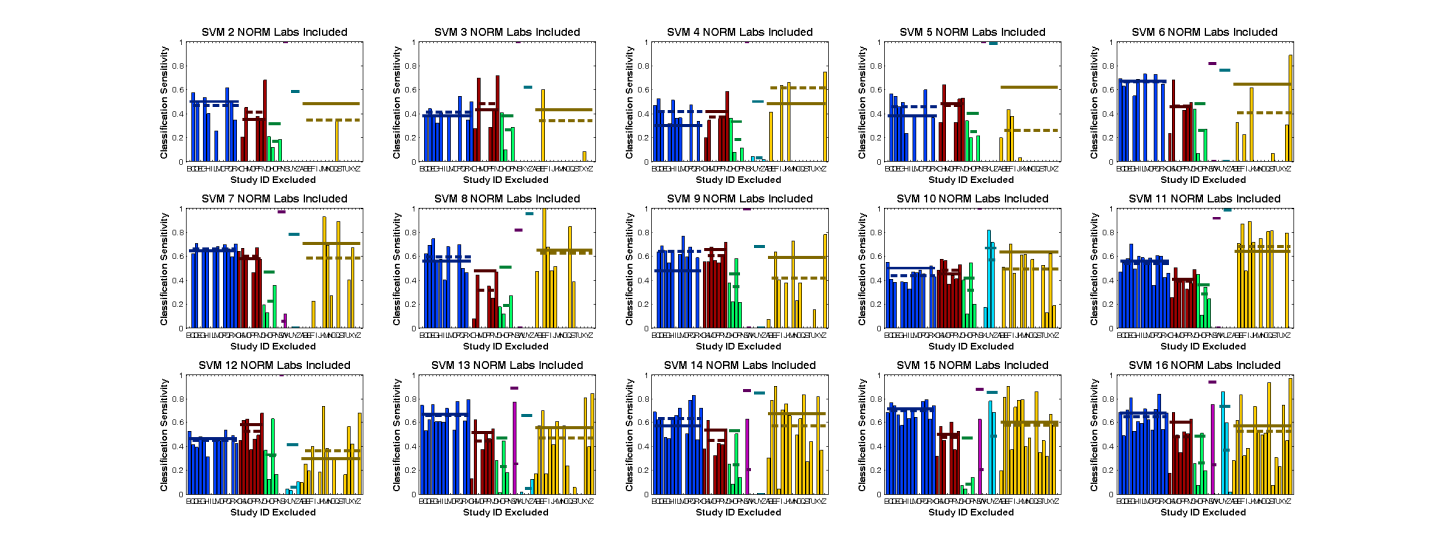


**Figure 12. Inter-study validation results varying NORM studies calculated by SVM.**


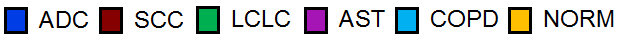


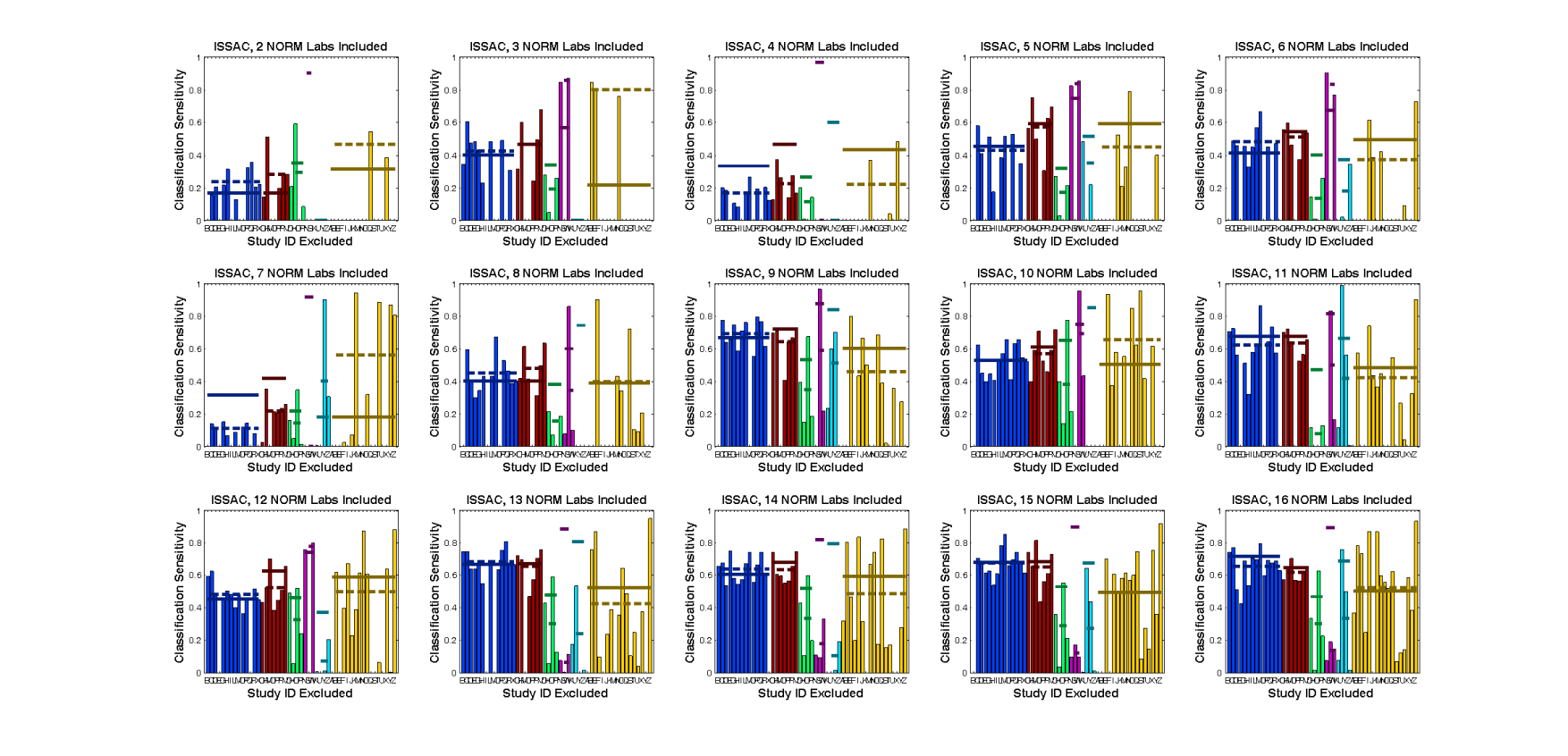


**Figure 13. Inter-study validation results varying NORM studies calculated by ISSAC.**

# Extended principal component analysis results

We identify the principal components that separated most effectively by phenotype. We find that the 3^rd^, 4^th^, and 5^th^, principal components capture more phenotype-associated expression variance than the 1^st^ or 2^nd^ principal components, which are largely affected by other sources of variance. In addition to examining by visual inspection (see Figure 14), we assess the extent to which samples of the same phenotype are grouped together by measuring the overlap of the phenotype groups with clusters selected using k-means clustering. We perform k-means clustering of the gene expression samples transformed into different pairs of principal component axes, setting *k=*6 (number of phenotypes in the analysis). For each phenotype, we find the fraction of samples that were labeled as cluster *k_i_* for each of the six clusters. We calculate this overlap for all of the phenotype-cluster combinations, and report the overlap accuracy of the phenotype-cluster mapping that maximizes the overlap averaged across phenotypes.


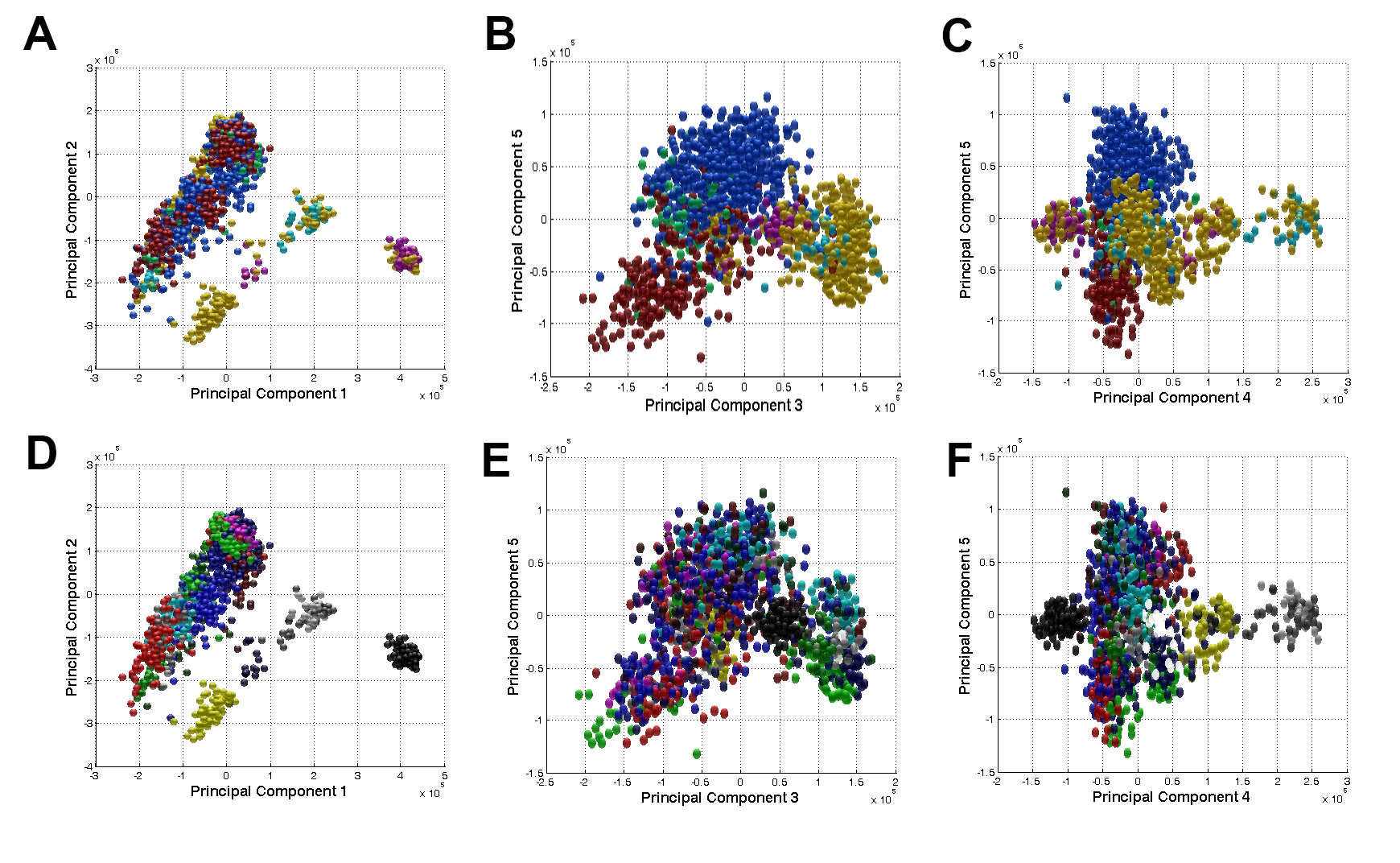

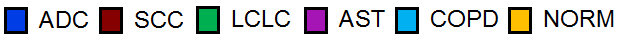


**Figure 14. Principal component analysis for the gene expression data examined in our study.** (A-C) Colors represent phenotype labels. (D-F) Colors represent study labels.

We find that when the data are represented by the 3^rd^ 4^th^ or 5^th^ principal components, the k-means clustering overlap is significantly greater than that when the data were represented by the other principal components (p < 0.05, t-test, see Figure 15). We find that the k-means overlap accuracy with phenotype clusters was 0.37 when data were projected onto the 1^st^ and 2^nd^ principal components. In contrast, the k-means overlap was 0.57 when data were onto the 3^rd^ and 5^th^ principal components and was 0.61 when projected onto the 4^th^ and 5^th^ principal components.


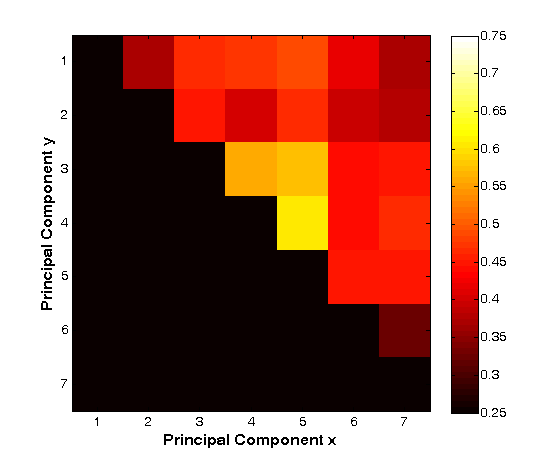


**Figure 15. K-means clustering overlap with phenotype labels as a function of principal component projection.** The colored squares represent the accuracy of overlap when the expression data are projected onto the two principal components represented by the x and y axes. The average overlap of k-means clusters and phenotype labels for data projected to 3^rd^ and 4^th^, 3^rd^ and 5^th^, or 4^th^ and 5^th^ principal components is 0.57; the average overlap for data not projected to these components is 0.43 (p < 0.05).

Leveraging the insight gained from PCA over all data considered in our study, we then compare the principal component representations of the data with different numbers of studies included for analysis (see Figure 16, Figure 17, and Figure 18). As we vary the numbers of considered experimental studies in a given phenotype (e.g., ADC), we compare the accuracy of overlap of the samples grouped by k-means clusters with the ADC samples in the PCA dataset, as quantified by the average of the fraction of ADC samples that were labeled as cluster *k** and the fraction of non-ADC samples that are not labeled as cluster *k*,* where *k** is the cluster that maximizes this overlap. We calculate this overlap for each of the phenotypes with which varied the number of studies included for analysis (i.e., ADC, SCC, and NORM).

We find that when the data are represented by the 1^st^ and 2^nd^ principal component axes, the k-means clustering overlap is negatively correlated with the increasing the number of ADC, SCC, and NORM studies included in the PCA dataset (Pearson correlation coefficient = -0.77, -0.78, and -0.77, respectively). In contrast, there is a positive correlation between the k-means clustering overlap and increasing the number of ADC and NORM studies when the 3^rd^ and 5^th^ principal component axes represent the data (correlation coefficient = 0.54 and 0.32, respectively) and a positive correlation with SCC when the 4^th^ and 5^th^ principal components are represented (correlation coefficient = 0.64). For each phenotype, when only two studies are included in the analysis, the samples belonging to that phenotype appear in close proximity with other phenotypes. As the number of studies incorporated into the analysis increases, the data appear to progressively shift toward a more separate, phenotype-specific cluster. These results suggest that including the number of studies in a meta-analysis strengthens the phenotype-associated signal in the gene expression.

To substantiate the implications of the k-means clustering overlap, we also assess the degree to which phenotypes are separable as the number of studies for a phenotype increases by examining the centroid separation distance. We calculate the Euclidean distance between the centroid of the samples belonging to the phenotype and the centroid of the samples not belonging to the phenotype as we varied the number of studies of the included in the analysis. We find that the first two principal components yield a negative correlation between the distance separating phenotype and not phenotype centroids and the number of phenotypes studies included when the phenotypes ADC, SCC, and NORM were considered. However, there is a significant positive correlation between the centroid distance separation and the number of studies included (p < 0.05 for each).


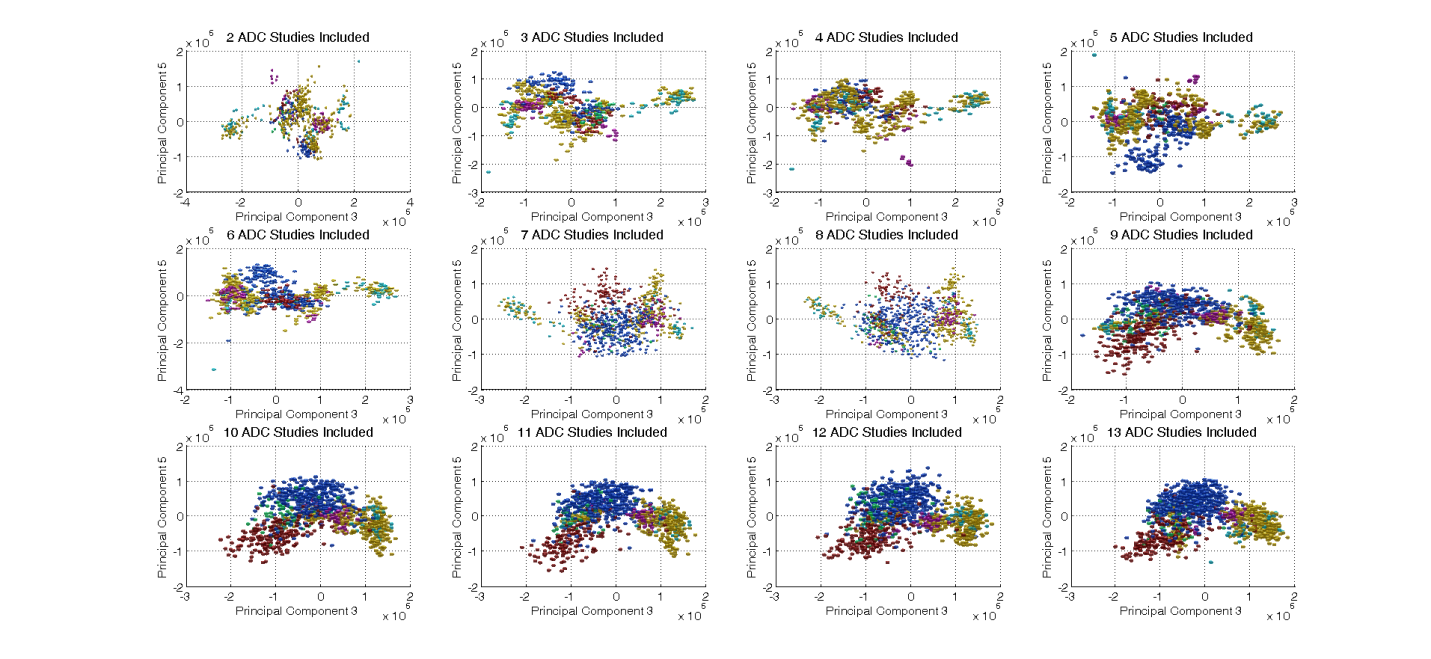

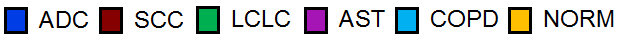


**Figure 16. Principal component analysis with varying numbers of ADC studies (blue).**


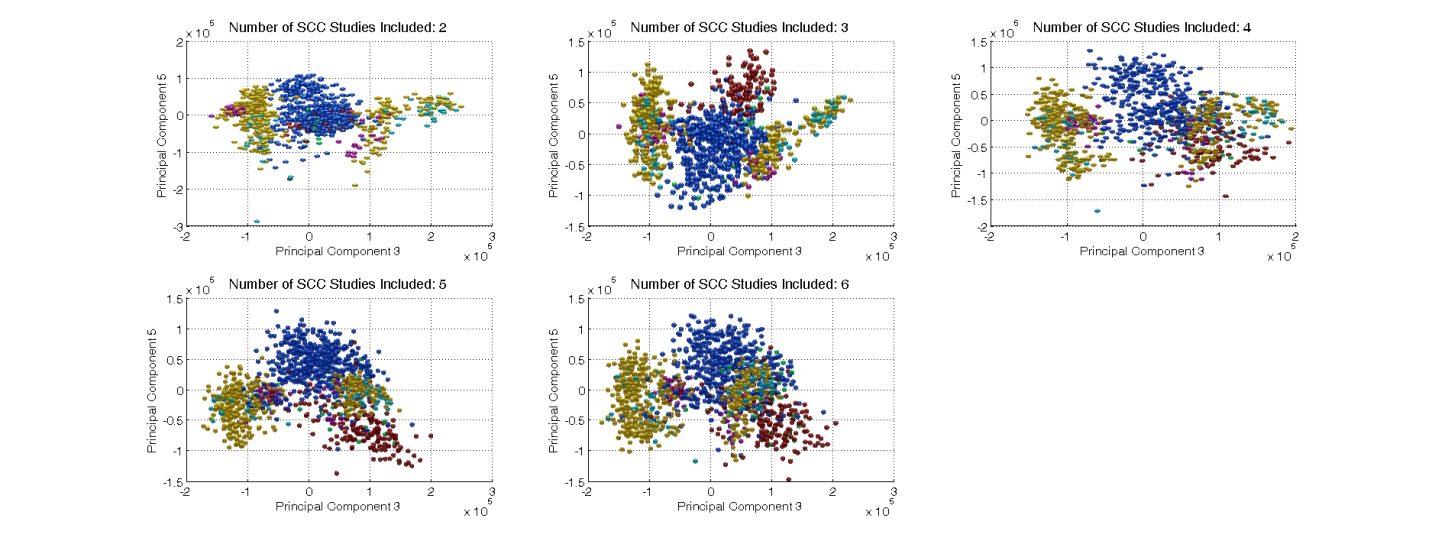

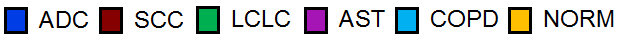


**Figure 17. Principal component analysis with varying numbers of SCC studies (red).**


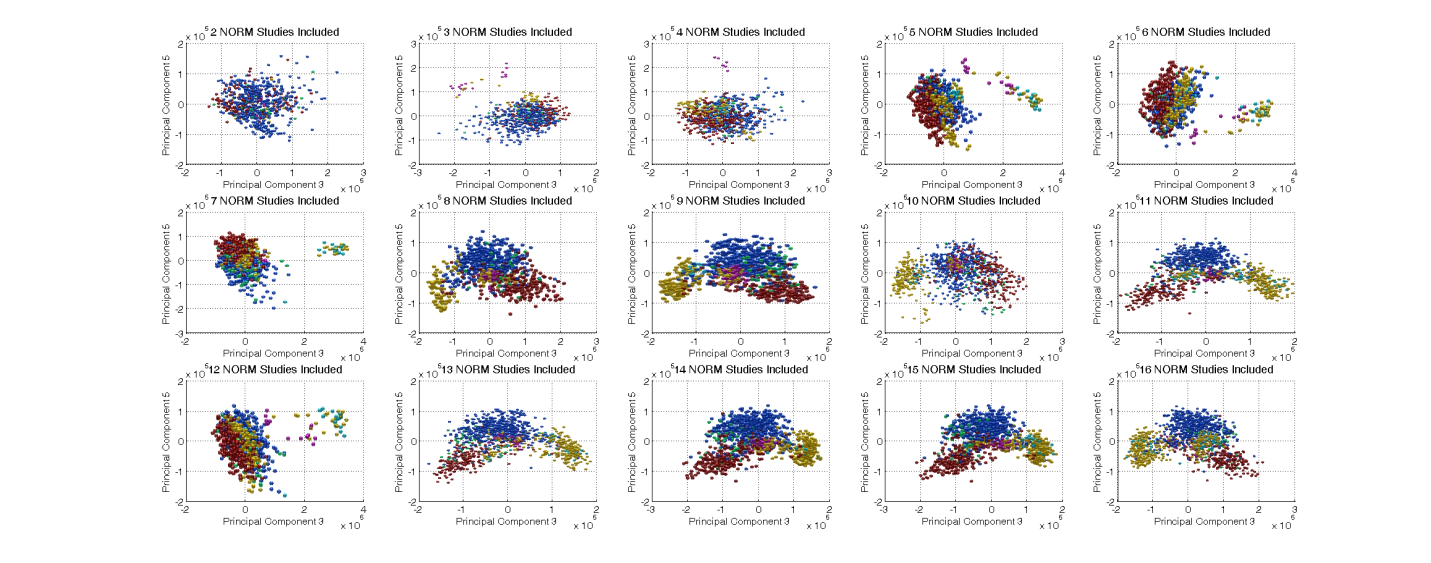

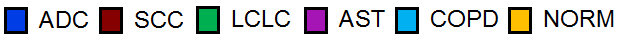


**Figure 18. Principal component analysis with varying numbers of NORM studies (yellow).**

# Impact of study-effects on the consistency of gene classifiers

We examined the feature genes selected via F-score for SVM and the ISSAC gene marker panels for the different iterations of cumulative CCVA. As we varied the number of ADC, SCC, or NORM studies included in the analysis, we observed some association in the consistency of the ISSAC gene marker panels and the F-score feature genes. We found that the number of ADC, SCC, or NORM studies included in CCVA was significantly correlated with the number of validation iterations in which the most consistent of the ISSAC marker panel genes appeared (Spearman’s rho > 0.61, p < 0.05). There was also a moderate but not significant correlation between the numbers of ADC, SCC, or NORM studies included in CCVA and the number of validation iterations in which the most consistent of the F-score feature genes appeared (Spearman’s rho ranged from 0.25 to 0.81 for the different phenotypes). This suggests that by introducing additional study-associated heterogeneity to mitigate the influence of study-effects on prediction error, we may also gain some benefit in improving classifier consistency.

# Description of disease classification algorithms used

## Support Vector Machines

The support vector machines method has been shown to classify phenotypes based on gene expression cases with high performance, and has also been amenable to classification in the context of feature selection methods [14-16]. The multiclass one-vs.one support vector machines (SVM) approach involves finding a series of maximum separating hyperplanes between samples from each pair of phenotypes, as defined by the multidimensional space defined by the expression of an input set of $f_{g}$ feature genes [14]. During prediction, each sample is tested against all of the classifying hyperplanes. The classification decision for each hyperplane is based on which side of the classifying hyperplane a sample’s expression profile falls [14]. The classifications from each of these hyperplanes are combined, and the overall prediction is the phenotype picked most frequently among the set of classifiers. We train the multiclass SVM using the LIBSVM library for MATLAB [17].

To control the number of input genes considered by SVM to construct each hyperplane, we further implement a feature selection algorithm, based on the F-score metric [16]. The F-score is calculated for each gene $i$ as [16, 18]:

,

where $j$ represents the samples included in the analysis, and $l$ represents the phenotypes to be classified. The $x_{\mathrm{ij}}$represented the expression of the *i*th gene in the $j$th sample, and the $y_{j}$ represented the phenotype class of the $j$th sample. The terms $\overline{x_{il}}$ and $\overline{x_{i}}$ represent the mean expression level for a gene within a phenotype and across all phenotypes, respectively. The $\delta(y_{j}=l)$ represents the Kronecker delta function:

$$\delta(y_{j}=l)=\left\{\begin{matrix} 1,y=l \\ 0,y\neq l \end{matrix}\right.$$

We perform feature selection based on the training data within each iteration of validation. To test the effect of the number of feature genes used in SVM on ISV and RCV performance, we compared the CCVA and cumulative CCVA results of SVM using the top $f_{g}$= 50, 500, and 5000 genes sorted by the F-score and found that the outcomes remained qualitatively comparable across these three selections for feature number (see Figure 19 and Figure 20). For convenience, we used the top $f_{g}$= 50 genes for the remaining analyses presented in the main text and supplemental information.


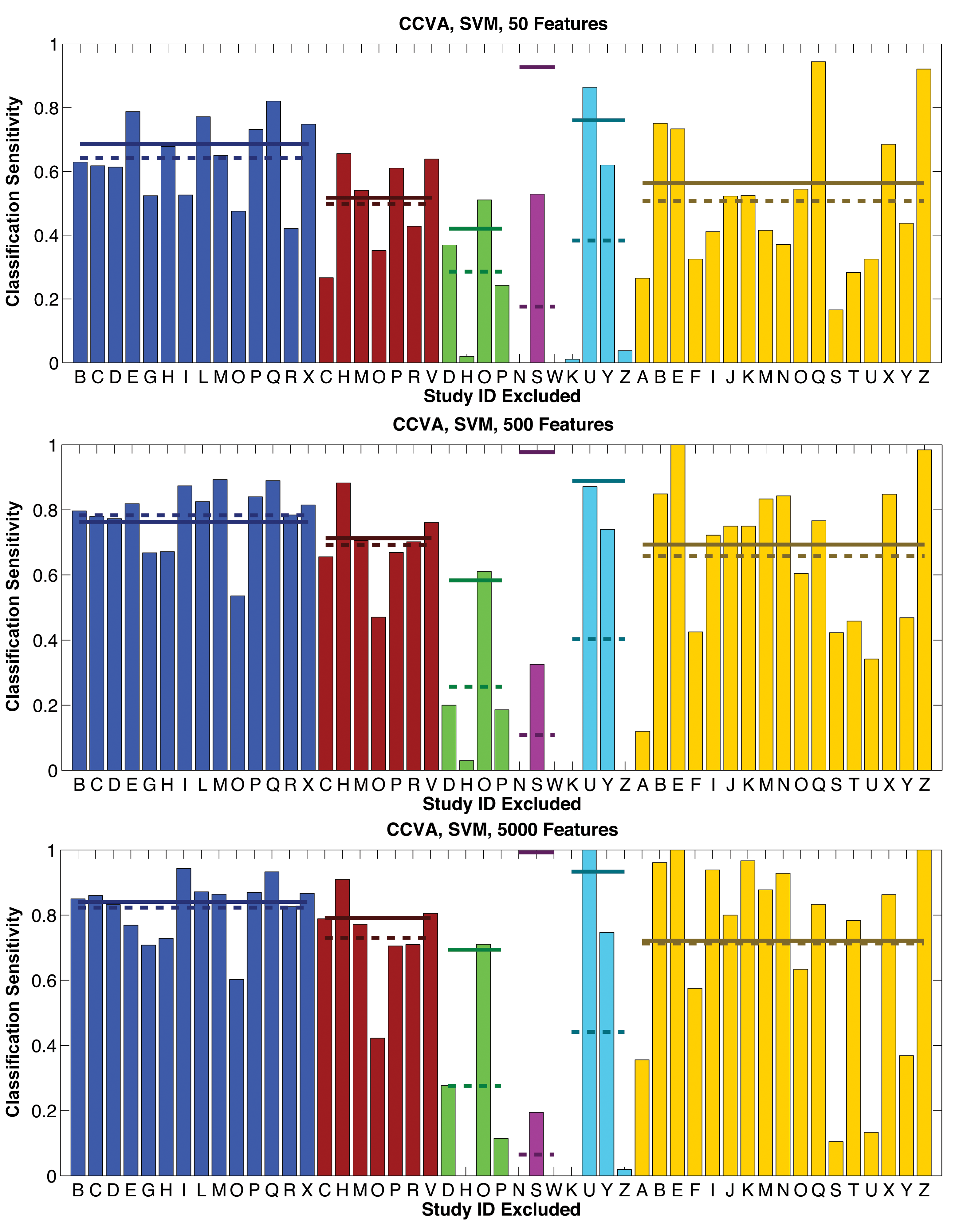

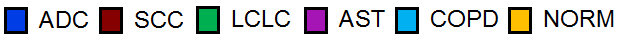


**Figure 19. Inter-study validation and randomized cross-validation performance calculated for SVM using 50, 500, or 5000 feature genes.** These plots are analogous to Figure 1a in the main text.

**
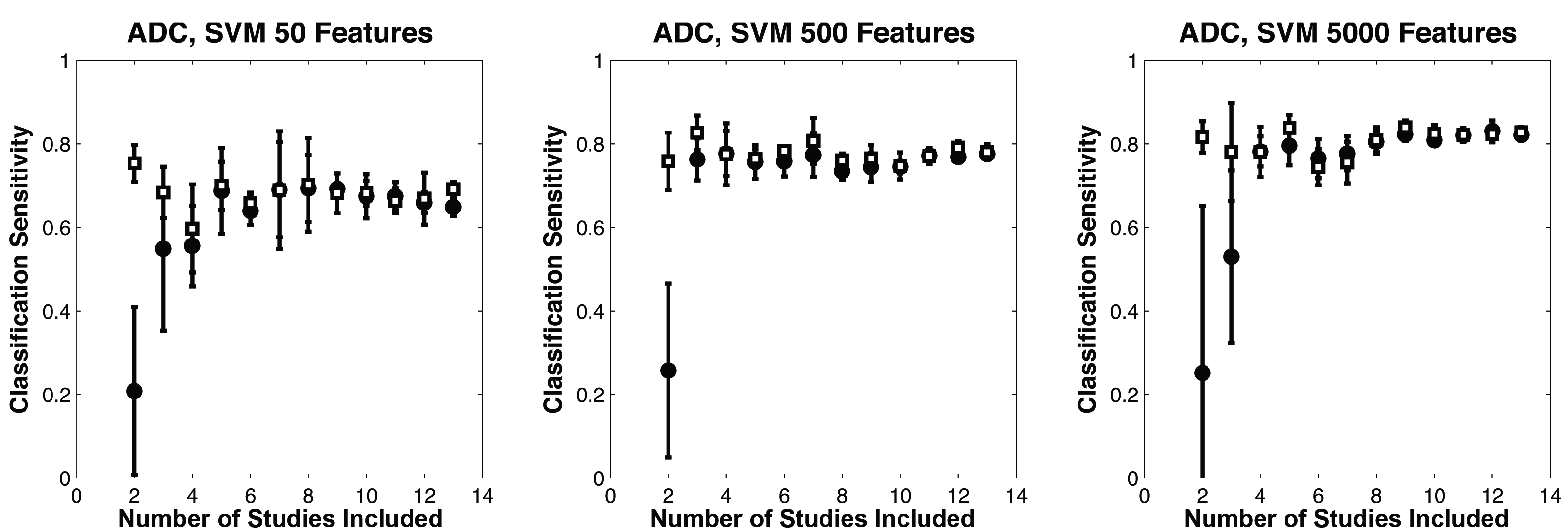
**


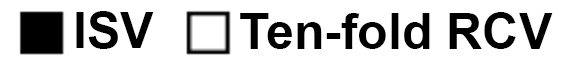


**Figure 20. Cumulative CCVA results for ADC calculated by SVM with 50, 500, or 5000 feature genes.** These plots are analogous to Figure 2a in the main text.

## Identification of Structured Signatures And Classifiers

The Identification of Structured Signatures And Classifiers (ISSAC) method [19] is a multiclass classification extension of the TSP learning algorithm [20], which predicts phenotypes based on the ranked expression levels of samples. To accommodate multiclass classification problems, ISSAC constructs a classification tree with an agglomerative hierarchical clustering approach, using the TSP score as the distance metric.

The classification tree informs two methods of sequential TSP comparisons. The first, node-based method focuses on the phenotype groups that comprise the nodes of each tree. A modified k-TSP method [21] is applied, yielding panels of gene pair classifiers to assess whether or not test samples belong to the phenotypes that are grouped at each node. If a sample is affirmatively classified, then it passes through both branches spanning from the node and faces classification at both downstream nodes until it reaches nodes with only one phenotype. If a sample is not classified as belonging to one of the phenotypes at a node, then it is rejected from further classification at downstream nodes. This node-based classification can make multiple predictions because a sample might pass classification at multiple branches in the tree. Furthermore, rejection of a sample from being classified by the tree indicates that the sample does not appear to resemble any of the phenotypes in the tree.

To break ties resulting from the node-based classification, a branch-based method is leveraged. The branch-based classification compares groups on each left branch against groups on the corresponding right branch with the binary TSP classification approach, yielding one gene pair classifier per branch. If a sample is classified as belonging to a phenotype in the left branch node, then it would subsequently be passed only down the branches that are downstream of the left node. Otherwise, the sample would be passed down for subsequent classification at the branches downstream of the right node, akin to a decision tree with the binary TSP scheme as the decision metric.

## Training set selection in validation

The number of microarray samples that belong to the six lung phenotypes varied substantially, ranging from 49 LCLC samples to 580 ADC samples. To mitigate biased classifiers resulting from skewed training sample sizes, we implement for the microarray evaluation a training data subset selection step within each iteration of validation. For each iteration of validation, we compare the samples sizes of each of the phenotypes represented in the portion of the data not excluded for testing purposes. The number of samples of the phenotype with the fewest samples, *n_low_*, is used as a benchmark. Phenotypes with sample numbers at least two times *n_low_* are evenly partitioned into disjoint random subsets of size comparable to *n_low_*. These partitions are then randomly selected to be included in data used to train the classifier. To ensure that classifier selection was not biased by the selection of the partitions, the training of the classifier is performed 10 times with independent selections of training data subset for each iteration of test set. Therefore, the performance of 10-fold randomized cross-validation with this training set selection is averaged from classifiers learned from 100 independent training subsets that are tested on 10 different test sets.

# References

1. Hubbell E, Liu WM, Mei R: **Robust estimators for expression analysis.** *Bioinformatics* 2002, **18:**1585-1592.

2. Irizarry RA, Hobbs B, Collin F, Beazer-Barclay YD, Antonellis KJ, Scherf U, Speed TP: **Exploration, normalization, and summaries of high density oligonucleotide array probe level data.** *Biostatistics* 2003, **4:**249-264.

3. Wu Z, Irizarry RA, Gentleman R, Martinez-Murillo F, Spencer F: **A model-based background adjustment for oligonucleotide expression arrays.** *Journal of the American Statistical Association* 2004, **99:**909-917.

4. Liu WM, Mei R, Di X, Ryder TB, Hubbell E, Dee S, Webster TA, Harrington CA, Ho MH, Baid J, Smeekens SP: **Analysis of high density expression microarrays with signed-rank call algorithms.** *Bioinformatics* 2002, **18:**1593-1599.

5. Wang Z, Gerstein M, Snyder M: **RNA-Seq: a revolutionary tool for transcriptomics.** *Nat Rev Genet* 2009, **10:**57-63.

6. Marioni JC, Mason CE, Mane SM, Stephens M, Gilad Y: **RNA-seq: an assessment of technical reproducibility and comparison with gene expression arrays.** *Genome Res* 2008, **18:**1509-1517.

7. t Hoen PA, Friedlander MR, Almlof J, Sammeth M, Pulyakhina I, Anvar SY, Laros JF, Buermans HP, Karlberg O, Brannvall M, et al: **Reproducibility of high-throughput mRNA and small RNA sequencing across laboratories.** *Nat Biotechnol* 2013, **31:**1015-1022.

8. Kim SC, Jung Y, Park J, Cho S, Seo C, Kim J, Kim P, Seo J, Park S, Jang I, et al: **A high-dimensional, deep-sequencing study of lung adenocarcinoma in female never-smokers.** *PLoS One* 2013, **8:**e55596.

9. Ju YS, Lee WC, Shin JY, Lee S, Bleazard T, Won JK, Kim YT, Kim JI, Kang JH, Seo JS: **A transforming KIF5B and RET gene fusion in lung adenocarcinoma revealed from whole-genome and transcriptome sequencing.** *Genome Res* 2012, **22:**436-445.

10. **Comprehensive genomic characterization defines human glioblastoma genes and core pathways.** *Nature* 2008, **455:**1061-1068.

11. Mailman MD, Feolo M, Jin Y, Kimura M, Tryka K, Bagoutdinov R, Hao L, Kiang A, Paschall J, Phan L, et al: **The NCBI dbGaP database of genotypes and phenotypes.** *Nat Genet* 2007, **39:**1181-1186.

12. Leinonen R, Sugawara H, Shumway M: **The sequence read archive.** *Nucleic Acids Res* 2011, **39:**D19-21.

13. Dobin A, Davis CA, Schlesinger F, Drenkow J, Zaleski C, Jha S, Batut P, Chaisson M, Gingeras TR: **STAR: ultrafast universal RNA-seq aligner.** *Bioinformatics* 2012, **29:**15-21.

14. Vapnik VN: **An overview of statistical learning theory.** *Ieee Transactions on Neural Networks* 1999, **10:**988-999.

15. Ben-Hur A, Ong CS, Sonnenburg S, Scholkopf B, Ratsch G: **Support vector machines and kernels for computational biology.** *PLoS Comput Biol* 2008, **4:**e1000173.

16. Chen YW, Lin CJ: **Combining SVMs with various feature selection strategies.** In *Feature Extraction Foundations and Applications.* *Volume* 207. Edited by Guyon I, Nikravesh M, Gunn S, Zadeh LA: Springer; 2006: *Studies in Fuzziness and Soft Computing*].

17. Chang CC, Lin CJ: **LIBSVM: a library for support vector machines.** In *Book LIBSVM: a library for support vector machines* (Editor ed.^eds.). City; 2001.

18. Mundra P, Rajapakse J: **F-score with Pareto Front Analysis for Multiclass Gene Selection.** *Evolutionary Computation, Machine Learning and Data Mining in Bioinformatics* 2009**:**56-67.

19. Sung J, Kim P-J, Ma S, Funk C, Magis A, Wang Y, Hood L, Geman D, Price N: **Multi-study Integration of Brain Cancer Transcriptomes Reveals Organ-Level Molecular Signatures.** *PLoS Comput Biol* 2013, **9**.

20. Geman D, d'Avignon C, Naiman DQ, Winslow RL: **Classifying gene expression profiles from pairwise mRNA comparisons.** *Stat Appl Genet Mol Biol* 2004, **3:**Article19.

21. Tan AC, Naiman DQ, Xu L, Winslow RL, Geman D: **Simple decision rules for classifying human cancers from gene expression profiles.** *Bioinformatics* 2005, **21:**3896-3904.
